# A Preliminary Study Introducing Electronic Patient-Reported Outcome (ePRO) Using Bring Your Own Device (BYOD) in Post-marketing Surveillance in Japan

**DOI:** 10.1007/s43441-025-00873-0

**Published:** 2025-09-24

**Authors:** Naomi Sugimoto, Mika Morimasa, Hidetoshi Misawa, Nobushige Matsuoka, Yurami Sato, Hiromi Yamaguchi, Tetsuya Hiraiwa, Natsuno Yamashita, Akira Hoshino, Masanori Kawai

**Affiliations:** 1 PMS Affairs, Pfizer R&D Japan, Shinjuku Bunka Quint Bldg. 3-22-7, Yoyogi, Shibuya-ku, Tokyo, 151-8589 Japan; 2 Biometrics & Data Management, Pfizer R&D Japam, Tokyo, Japan; 3 Pfizer Digital, Pfizer Japan Inc., Tokyo, Japan

**Keywords:** Bring your own device, BYOD, Patient-reported outcome, ePRO, Post-marketing surveillance, Observational study

## Abstract

**Background:**

The method of collecting electronic patient-reported outcome (ePRO) data using the bring your own device (BYOD) approach has become very common recently, especially in clinical trials. We conducted a preliminary study to evaluate the processes before introducing ePRO using BYOD in regulatory-required observational post-marketing surveillance (PMS) in Japan.

**Methods:**

We conducted a multicenter observational study using a two-period, two-sequence, cross-over design. Participants were allocated to Group 1 (starting with ePRO) or Group 2 (starting with paper PRO). The observation period was 14 days in total: seven days each for ePRO and paper PRO. We assessed the usability, implementation process, support system, and materials for ePRO through questionnaires.

**Results:**

All participants in Group 1 (n = 78) and Group 2 (n = 73) were included in the analysis set. The collection of information with ePRO was comparable to that with paper PRO. We found no remarkable difference in data entry status between ePRO and paper PRO based on sex and no trend toward a higher proportion of missing data in ePRO with age. More than half of the participants responded favorably to most of the questionnaire items about ePRO. Although the investigators considered the materials for the ePRO system useful, there is still room for improvement.

**Conclusion:**

Our two-week pilot indicates that ePRO can achieve data completeness comparable to paper in motivated clinics. The points to consider when using ePRO in actual PMS were confirmed. Further assessment in actual studies conducted in compliance with local regulations is needed.

**Supplementary Information:**

The online version contains supplementary material available at 10.1007/s43441-025-00873-0.

## Introduction

Collecting patient-reported outcome (PRO) data is often crucial in clinical research to assess the benefits of medical products from the patient’s perspective [1, 2]. While PRO data were historically collected on paper, there has been a recent increase in using electronic PRO (ePRO), such as on smartphone or tablet devices, to collect this information. Capturing ePRO offers several advantages, including (1) more accurate, complete, and contemporaneous data with date and time stamps to ensure and demonstrate their timeliness; (2) avoidance of secondary data entry errors; (3) easier implementation of skip patterns; (4) high respondent acceptance; (5) improved trial protocol compliance; (6) increased power of statistical analyses and possible associated reduction in sample size requirements; and (7) less administrative burden [3]. The bring your own device (BYOD) approach, where study participants use their own devices for ePRO, can offer several advantages to participants, sites, and sponsors compared to using provisioned devices, despite operational challenges [4, 5]. For example, BYOD can reduce the study sponsors’ costs and time spent on purchasing, maintaining, shipping, and distributing devices to sites/participants. For study sites, the difficulty in managing and storing devices and training participants on the use of devices can be reduced. Participants benefit from using familiar devices, potentially reducing the bias with new devices and avoiding the need to manage another device just for ePRO completion.

In Japan, ePRO has become more common for PRO data collection in clinical trials [6], but it is not widely used in post-marketing surveillance (PMS) studies. PMS studies are planned as additional pharmacovigilance activities under the Japan Risk Management Plan (J-RMP) for each pharmaceutical product and are conducted under the local law called Act on Pharmaceuticals and Medical Devices and the ordinance called Good Post-marketing Study Practice (GPSP) [7]. Drug use-results survey (DUS) is a type of PMS that collect data from participants at investigational sites in real clinical settings. The need to collect PRO information in a DUS may be limited (e.g., records of topical and systemic reactions after vaccination in participant diaries), and regardless of whether BYOD is used, ePRO is currently not as commonly used as in clinical trials. This could be due to the difference between DUSs and clinical trials. Clinical trial participants are highly motivated to actively participate in studies of an investigational drug that has not reached the market and may even be willing to use an ePRO if required. In contrast, DUS participants do not have the motivation to participate in studies for a marketed drug and may not appreciate the benefit of using ePRO. Moreover, without the support of a research coordinator, which is typical of clinical trials, investigators may find familiarizing themselves with ePRO systems and processes and explaining them to participants to be an additional burden. However, ePRO using the BYOD approach can provide similar benefits to a DUS as in a clinical trial.

Thus, we conducted a preliminary study in preparation for implementing ePRO using BYOD in a real DUS as proof-of-concept. The objective of this study was to evaluate the processes for introducing ePRO using BYOD, including data linkage between Electronic Data Capture (EDC) and ePRO. PRO data was also collected using a paper-based observational diary (paper PRO). The study also assessed the data collection status of ePRO and paper PRO, as well as the usability of ePRO, including the implementation process, support systems, and materials provided.

## Materials and Methods

### Study Design and Participants

**Fig. 1:**
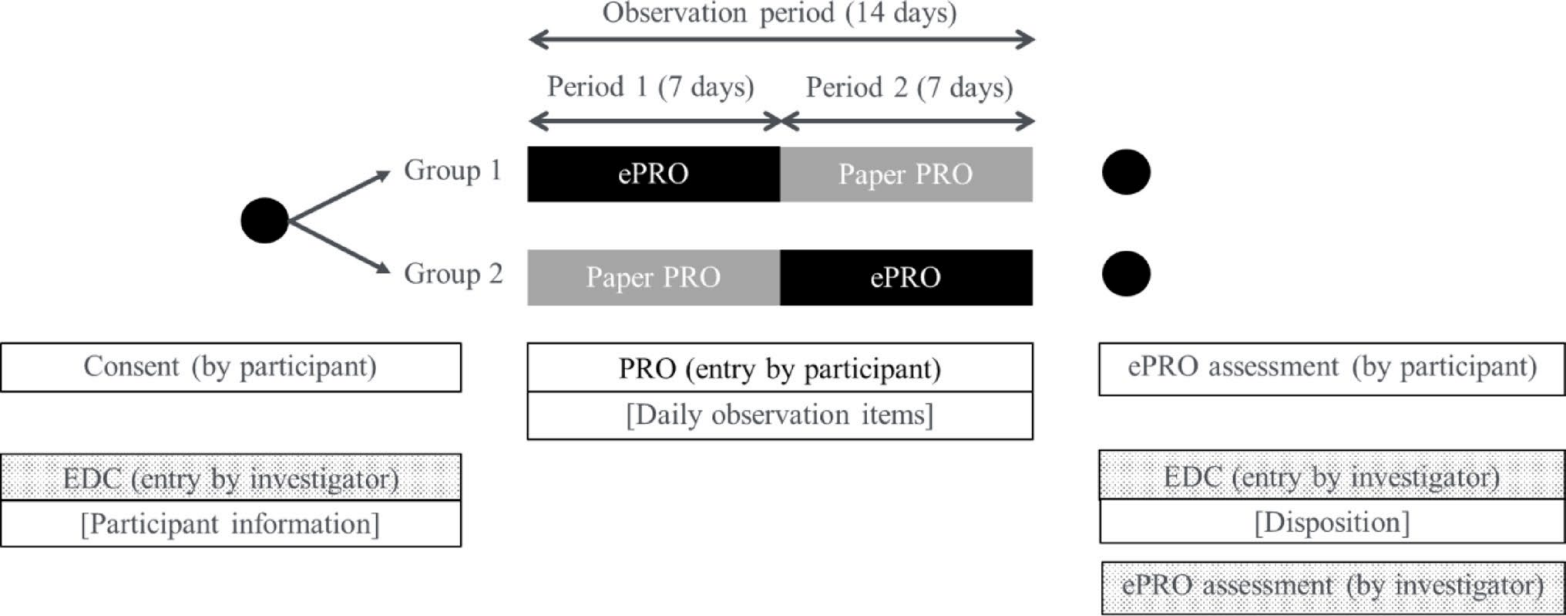
Study design. EDC, electronic data capture; PRO, patient-reported outcome

This multicenter observational study was conducted in a two-period, two-sequence, cross-over design (Fig. 1). Participants were allocated to two groups: one group entered information using ePRO (Supplementary Material 1) in the first half (7 days) of the observation period (Period 1) and completed paper PRO (Supplementary Material 2) in the latter half (7 days) (Period 2) (Group 1: ePRO to paper PRO), while the other group entered information using paper PRO in Period 1 and completed ePRO in Period 2 (Group 2: paper PRO to ePRO). Specifically, investigators assigned participants based on the allocation group enclosed in sealed opaque envelopes following the order of enrollment. Those envelopes with enrollment sequence numbers were prepared by the sponsor (Pfizer) and distributed to each investigational site. The observation period lasted 14 consecutive days, with 7 days each for ePRO and paper PRO, starting on the day the participant agreed to participate and began the study, as Day 1.

Patients who visited the investigational sites and met the inclusion criteria below were enrolled as study participants:

1. Patients who could understand the study content and enter their own information in the ePRO system using their own devices (smartphone, tablet, etc.) and in paper PRO,
2. Patients having a stable disease status and who could be expected to visit the site for a certain period, and
3. Patients who provided written consent to participate in this study and for the publication of the results.

Although not strictly specified, rough guidance for enrollment in each site was set at 50% for females and 25% for those aged 65 years or older. There were no exclusion criteria.

The target number of participants was set to 200 based on the feasibility during the study period from December 2022 to June 2023 (enrollment period was from December 2022 to March 2023).

### Study Procedure

The data collection tool and persons responsible for entering each investigation item are listed in Table 1. Various types of data, such as numerical, categorical, and ordinal data, were collected in both ePRO and paper PRO to evaluate the collection of data with different characteristics. Disease and treatment information was not collected in this study.

**Table 1:** Data collection tool and entry for each investigation item

Investigation item	Details
Participant information (registration form/CRF)	Eligibility for enrollment, identification number, sex, birth year and month (or age), observation start date (date when the participant consented to start the study at the visit), allocated group (Group 1: ePRO to paper PRO, Group 2: paper PRO to ePRO)
Observation items (diary)	Observation date (yyyy/mm/dd), time of awakening [hh (hours): mm (minutes)], sleep state of previous day (choose one from four options), number of meals (including snacks), general appetite (choose present or absent), time spent for going out (hh: mm), time spent for exercise (choose one from three options), fatigue before bedtime (choose one from four options), and drinking (alcohol consumption: choose present or absent)
Record of completion of observation* (CRF)	Completed/reason for not completion
ePRO assessment	Usability and implementation process of ePRO, satisfaction with the training material provided, support system such as helpdesk, etc

CRF: case report form, EDC: electronic data capture, PRO: patient-reported outcome

^*^Record of completion of observation. Of the enrolled participants, those who had at least one data point in each of the three forms (ePRO, paper PRO, and ePRO assessment) were assessed as having completed observations.

Investigators enrolled participants in the EDC system on the day of consent and sent them an e-mail invitation to the ePRO system. The participants set up their accounts on the ePRO system using their own devices immediately after receiving the invitation e-mail; they could enter the data in ePRO after completing e-learning.

Data entered in the ePRO system by a participant were automatically linked to the EDC in real time.

Data entry in the ePRO system was allowed for 48 hours starting at the beginning of each observation day. It was designed such that it would no longer be valid after the due date to ensure immediacy and reliability. Data entry with blank fields was not allowed, and the system was designed such that no partially missing data occurred. An automatic e-mail alert sent by the ePRO system encouraged participants to minimize missed data entries.

Data in paper PRO were submitted to Pfizer through the investigational sites after completing the 7-day observation period, and entered into the EDC by Pfizer. Data recorded by the next day of each observation date were eligible.

All information collected from participants using ePRO and paper PRO was integrated into the EDC system, allowing the investigators to review and evaluate the information collected from participants on the EDC. The data collected by ePRO and paper PRO were accepted for analysis without seeking any clarification and determined after some adaptation as needed.

The usability, including implementation process, support system, and materials for ePRO, were assessed using questionnaires to participants and investigators, including healthcare professionals.

myMedidata, a web-based patient portal, and Rave EDC are two solutions by Medidata Solutions that were used as data-capturing tools to collect patient information from investigational sites. myMedidata eCOA, an ePRO solution, was used to collect study information directly from participants. The EDC and ePRO system support was outsourced to Medidata Solutions, Inc. (New York), a Dassault Systèmes Company, and help desks were installed for both investigational sites and participants.

### Data Analysis

The primary analysis population included all participants who provided informed consent to participate in the study and publish the study results and who were assigned to either Group 1 (ePRO to paper PRO) or Group 2 (paper PRO to ePRO), excluding those with registration violations.

To evaluate the completeness and accuracy of data collection from participants, missing data, duration of data entry, and unevaluable data were summarized by the PRO collection method throughout Period 1 and Period 2, and in each period. Unevaluable data were identified before the database release. Missing data occurred when no data were collected within the allowed timeframe (target day of observation and the next day). Unevaluable data collected outside the allowed timeframe were treated as “missing” rather than “unevaluable” data.

The proportion of participants with missing data was analyzed using generalized estimating equations (GEE) to fit a repeated-measures logistic regression with period, group (i.e., sequence) and PRO collection method as fixed effects, accounting for the correlation between the observations in periods 1 and 2 within participants. The exchangeable working correlation structure and the empirical variance estimator were used in the model. The odds ratio (relative to paper PRO) and its 95% confidence interval (CI) were calculated from the model.

As exploratory analyses, subgroup analyses by sex and age were conducted to explore the differences in data entry between subgroups.

During the first approximately one month, the automatic alert e-mails to inform data entry due date were not delivered due to a system error. Sensitivity analyses were conducted for participants, excluding participants affected by the error.

The participants’ and investigators’ responses to questionnaires on the usability, including implementation process, support system, and materials for ePRO, were descriptively summarized.

All analyses were pre-specified before the database release.

## Results

### Demographics of Participants

During the enrollment period, 151 participants who consented to participate in 15 sites were enrolled. The case report forms (CRFs) of all enrolled participants were collected and fixed by EDC, and all 151 participants were included in the analysis set. The target number was not achieved, but enrollment was completed because the number of evaluable participants required for the purpose of this study could be collected.

Table 2 presents the demographics of the participants. Of the 151 participants, 78 were allocated to Group 1 and 73 to Group 2. Approximately 60% of the participants were female, and approximately 25% were aged 65 years or older. The proportion of females was higher than that of males. Of the three participants aged below 20 years in age category 2, two were 18 years old, and one was 19 years old. The 52 participants aged 60 years or older included 33 in their 60s, 17 in their 70s, and 2 in their 80s. There were no major differences in mean age between Group 1 and Group 2. The proportion of older adult participants aged 65 or more and that of participants aged 20 or more were also similar in the two groups.

**Table 2:** Demographics of participants

	Group 1 (N = 78*)	Group 2 (N = 73*)	Total (N = 151*)
Sex	n (%)	n (%)	n (%)
Male	28 (35.9)	31 (42.5)	59 (39.1)
Female	50 (64.1)	42 (57.5)	92 (60.9)
Age			
Mean (SD)	50.0 (17.27)	51.6 (15.29)	50.8 (16.31)
Median (range)	49.5 (18–83)	55.0 (22–78)	53.0 (18–83)
Category 1	n (%)	n (%)	n (%)
< 65	58 (74.4)	56 (76.7)	114 (75.5)
≥ 65	20 (25.6)	17 (23.3)	37 (24.5)
Category 2	n (%)	n (%)	n (%)
< 20	3 (3.8)	0	3 (2.0)
≥ 20, < 30	11 (14.1)	8 (11.0)	19 (12.6)
≥ 30, < 40	9 (11.5)	11 (15.1)	20 (13.2)
≥ 40, < 50	16 (20.5)	8 (11.0)	24 (15.9)
≥ 50, < 60	11 (14.1)	22 (30.1)	33 (21.9)
≥ 60	28 (35.9)	24 (32.9)	52 (34.4)

Group 1: ePRO to paper PRO, Group 2: paper PRO to ePRO

*Participants who completed observation: Participants who had at least 1 data in each of 3 forms; ePRO, paper PRO, and ePRO assessment

### Proportion of Missing Data

**Fig. 2:**
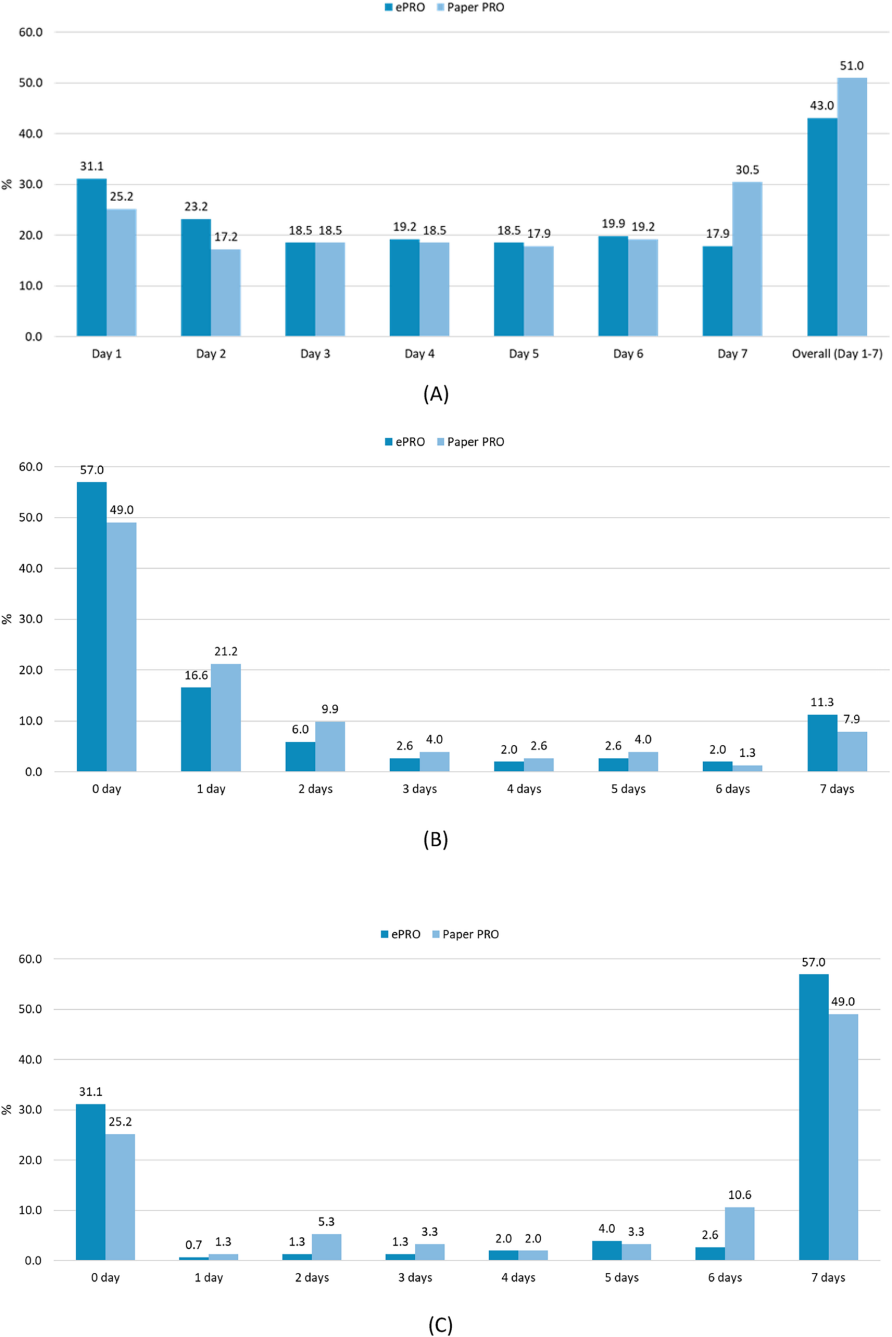
Comparison of missing data between ePRO and paper PRO. **A** Proportion of missing data. **B** Days with missing data. **C** Consecutive days of evaluable data

The odds ratio of the proportion of missing data for ePRO versus paper PRO is shown in Fig. 2A and Table 3. The proportion of missing data collected with ePRO was not higher than that with paper PRO in the overall period (Day 1 to Day 7) and was comparable to that with paper PRO. Missing data in all data items by time point were most frequently observed on Day 1 for ePRO (31.1%); this was observed on Day 7 (30.5%) and Day 1 (25.2%) for paper PRO. Although the odds ratios for Days 1 to 6 were > 1 except for Day 7 (0.49), the proportion of missing data with ePRO showed no clear tendency to be higher than that with paper PRO. Sensitivity analysis conducted on 105 participants, excluding 46 participants who had been affected by ePRO data entry alert e-mail delivery failure during the observation period, showed that the proportion of missing data was 38.1% (40/105 participants) for ePRO and the odds ratio against paper PRO was 0.58 (95% confidence interval: 0.35–0.96), indicating a lower proportion of missing data compared to that with paper PRO.

**Table 3:** Proportion of missing data by PRO collection method

Time point	ePRO (N = 151)	Paper PRO (N = 151)
	n (%)	n (%)
	Odds ratio* (95% confidence interval)	
Overall (Day 1—7)	65 (43.0)	77 (51.0)
	0.71 (0.47–1.09)	
Day 1	47 (31.1)	38 (25.2)
	1.36 (0.90–2.06)	
Day 2	35 (23.2)	26 (17.2)
	1.49 (0.93–2.40)	
Day 3	28 (18.5)	28 (18.5)
	1.00 (0.61–1.66)	
Day 4	29 (19.2)	28 (18.5)
	1.03 (0.61–1.75)	
Day 5	28 (18.5)	27 (17.9)
	1.04 (0.59–1.85)	
Day 6	30 (19.9)	29 (19.2)
	1.04 (0.61–1.77)	
Day 7	27 (17.9)	46 (30.5)
	0.49 (0.29–0.82)	

*Odds ratio of ePRO to paper PRO

**Fig. 3:**
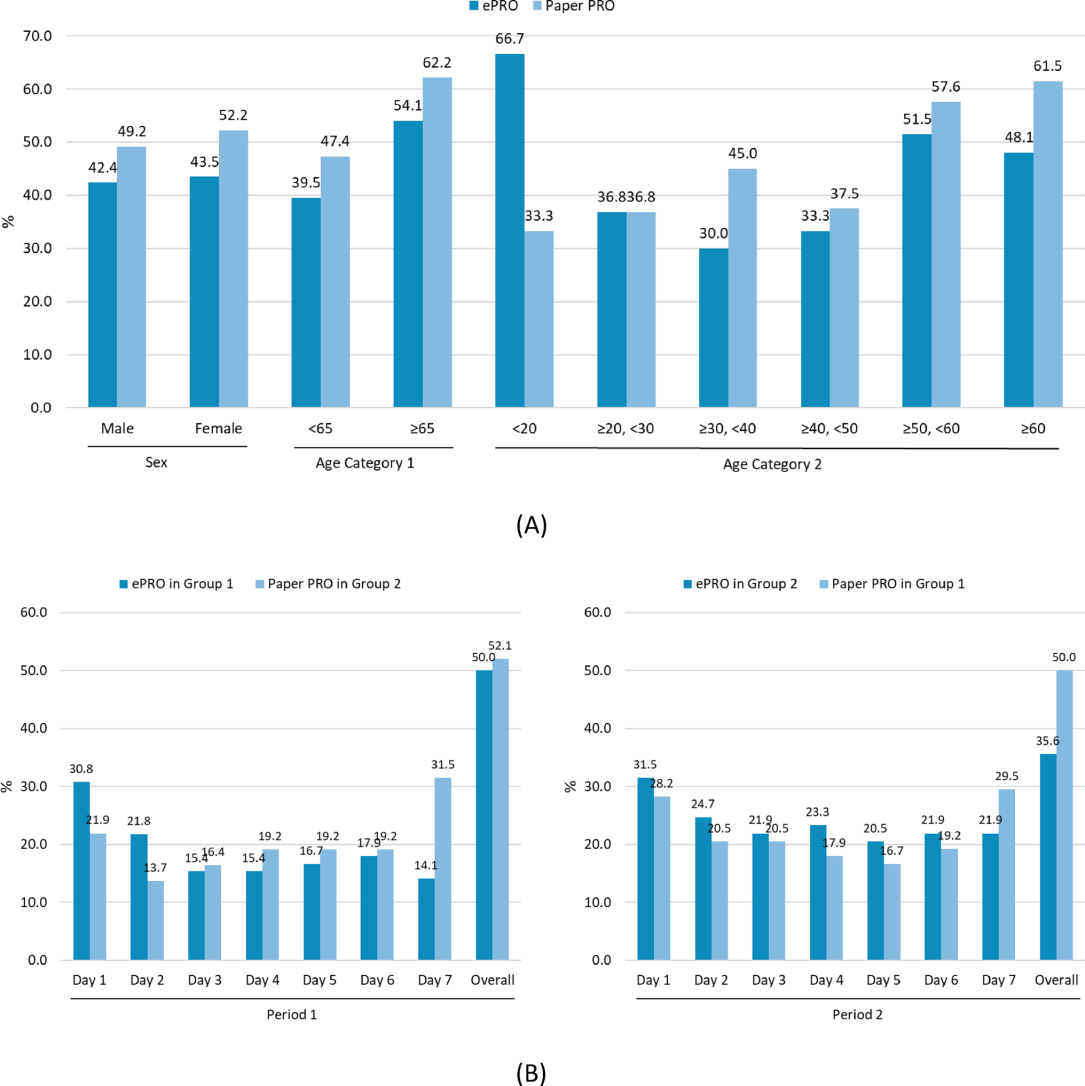
Proportion of missing data by PRO collection method: by subgroup and period. **A** By subgroup of Sex, Age. **B** By period

Subgroup analysis (Fig. 3A and Supplementary Table S1) showed almost similar proportions of missing data (all items, overall period) with ePRO and paper PRO for males and a slightly higher proportion with paper PRO than ePRO for females. No major differences were observed in the proportion of missing data by sex. The proportion of missing data with paper PRO was higher than that with ePRO for both the non-older adults (< 65) and older adults (≥ 65). Furthermore, the missing proportion was higher for older adults compared to non-older adults with both ePRO and paper PRO. By age group, the proportion of missing data with paper PRO was higher for all age groups in their 30s and above.

The differences in the proportion of missing data in Period 1 (seven days, first half of the observation period) and Period 2 (latter half) were assessed for Group 1 (started with ePRO) and Group 2 (started from paper PRO) (Fig. 3B and Supplementary Table S2). During the overall period, there was no major difference between ePRO and paper PRO for all items in Period 1 (from Day 1 to 7) for Group 1 and Group 2. However, a lower proportion of missing data was observed with ePRO compared to paper PRO in Period 2. Although the proportion of missing data with ePRO in Period 1 (Group 1) was initially higher than that in Period 2 (Group 2), it decreased notably from Day 2 onward. In Group 2, which used ePRO in Period 2, the proportion of missing data from Day 2 onward was stable at approximately 20%. Conversely, no major difference was found in the proportion of missing data with paper PRO regardless of period (Period 1 or Period 2) or sequence (Group 1 or Group 2). Sensitivity analysis conducted on 105 participants to assess the effect of the ePRO input alert e-mail delivery failure showed that although the proportion of missing data was generally lower than that for all participants, there was no major difference in the trend across periods or sequences.

### Days with Missing Data

In the analysis of days having missing data (i.e., the occurrence of missing data on each day in each period), unevaluable data were not considered missing. For the 151 participants in the analysis set, the mean ± SD of days with missing data was similar between ePRO and paper PRO (Fig. 2B and Supplementary Table S3). However, the proportion of participants with no missing data (0 days) was higher with ePRO than paper PRO. Furthermore, sensitivity analysis conducted on 105 participants to assess the effect of ePRO input alert e-mail delivery failure revealed no major difference compared to all participants.

Subgroup analysis (Supplementary Table S4) showed similar results for males and females. The mean number of days of missing data with ePRO was similar to that with paper PRO for the non-older adults. However, it was slightly higher than that with paper PRO for older adults. The mean number of days of missing data with ePRO was higher for older adults than non-older adults. The mean number of days with missing data was lowest for the 30s age group and highest for the 50s age group.

There were no major differences in the proportion of missing data across periods and sequences (Supplementary Table S5).

### Consecutive Days of Evaluable Data

The evaluation of continuous data entry from Day 1 (Fig. 2C and Supplementary Table S6) revealed that the mean ± SD of consecutive days was almost the same between ePRO and paper PRO, with seven days being the most common duration for both, followed by 0 days. Most participants either maintained consistent data entry or completed the study without any entry. Thus, a low proportion of participants discontinued the study during the observation period. Sensitivity analysis with 105 participants to assess the impact of ePRO input alert e-mail delivery failure indicated similar mean ± SD of consecutive days for ePRO and paper PRO, with a higher proportion of participants maintaining data entry for longer durations compared to all participants. Subgroup analysis (Supplementary Table S7) indicated no major differences by sex, although consecutive days with ePRO were slightly shorter than with paper PRO among older adults, with the older adults showing one day less compared to the non-older adults. Notably, consecutive days with ePRO did not differ from paper PRO in each age group, with the mean duration being shortest in the 50s group and no tendency to shorten with age.

No major difference in means was observed between Period 1 and Period 2, as well as between Group 1 and Group 2 (Supplementary Table S8). Following a sensitivity analysis involving 105 participants to evaluate the impact of ePRO input alert e-mail delivery failure, no major difference could be found compared to all participants.

### Unevaluable Data

There were very few instances of unevaluable data for the entire observation period, with five cases (3.7%) recorded with ePRO and four cases (2.9%) with paper PRO. These data were observed in “time spent for going out” with ePRO, and “time spent for exercise” and “drinking” with paper PRO. The data observed in “time spent for going out” with ePRO that could not confirm whether it was entered by minutes or a colon was missed were determined as unevaluable. For “time spent for exercise” and “drinking,” cases in which multiple choices were selected despite instructions to choose only the closest option from the selections were determined to be unevaluable in paper PRO. Notably, the ePRO system did not allow for multiple selections.

### ePRO Usability Assessment by Participants and Investigators

Table 4 and Supplementary Table S9 presents the participants’ assessment results. Approximately 65% to 70% of responses were favorable for invitation e-mail and account activation (Q1 and 2), whereas approximately 10% to 15% responded unfavorably. Similar trends were observed for materials provided for participants (Q3 and 4). Approximately 70% of participants found data entry in ePRO easy, as indicated by favorable responses to Q5, whereas approximately 15% responded unfavorably. Approximately 60% of participants expressed that they can enter data into ePRO without any issues in the future (Q8), with approximately 20% responding unfavorably. Subgroup analysis of Q8 by age group showed no tendency for older adult participants to avoid electronic data entry. Less than half of the participants found alert e-mails helpful for preventing missing entries (Q6). Approximately 70% of participants did not contact the myMedidata help desk, and the responses of those who did inquire varied (Q7).

**Table 4:** Summary of ePRO usability assessment by participants

Question		Group 1: n/N (%)	Group 2: n/N (%)	Total: n/N (%)
Q1. Was e-mail invitation for ePRO (myMedidata) received easily?				
	Favorable (very much or somewhat)*	53/78 (67.9)	45/71 (63.4)	98/149 (65.8)
Q2. Was account activation for ePRO (myMedidata) completed smoothly?				
	Favorable (very much or somewhat)	49/74 (66.2)	46/61 (75.4)	95/135 (70.4)
Q3. Was the material “myMedidata patient registration workflow (for participants)” easy to understand?				
	Favorable (very much or somewhat)	47/74 (63.5)	42/60 (70.0)	89/134 (66.4)
Q4. Was the material “Data entry manual for ePRO (myMedidata)” easy to understand?				
	Favorable (very much or somewhat)	46/74 (62.2)	49/60 (81.7)	95/134 (70.9)
Q5. Was data entry in ePRO (myMedidata) easy?				
	Favorable (very much or somewhat)	52/73 (71.2)	44/59 (74.6)	96/132 (72.7)
Q6. Was e-mail alert for data entry in ePRO (myMedidata) helpful in avoiding missing entry? (Only participants not affected by delivery failures of alert e-mail were involved in analysis set.)				
	Favorable (very much or somewhat)	18/47 (38.3)	24/41 (58.5)	42/88 (47.7)
Q7. Was the issue resolved within the expected timeframe at “Medidata helpdesk (Patient Cloud Device Support)”?				
	Favorable (very much or somewhat)	5/73 (6.8)	9/57 (15.8)	14/130 (10.8)
Q8. Based on experience in this study, will electronic data entry by devices such as smartphones, PCs, etc. be able to be done without issue, if you have another opportunity to record your health information in the future?				
	Favorable (very much or somewhat)	45/70 (64.3)	34/59 (57.6)	79/129 (61.2)

Number of participants: Group 1; 78, Group 2; 73, Total; 151

There were participants who did not receive invitation (Q1), complete account activation (Q2), enter any data (Q5, 6), receive alert (Q6) and make inquiry (Q7). (See Supplementary Table S9)

*Assessed in 5 grades: Very much / Somewhat / Neutral / Not so much / Not at all

Table 5 and Supplementary Table S10 presents the investigators’ assessment results. Approximately 60% of responses on the usefulness of the training material for ePRO were favorable (Q4). However, as regards its understandability, favorable, neutral, and unfavorable responses showed similar levels (Q3). While approximately 60% found the material provided to explain ePRO use (informed consent document) useful (Q6), approximately 40% of respondents found it difficult to understand (Q5). The smoothness of the explanation for ePRO use (Q7) yielded controversial responses. There were over 60% of neutral or unfavorable responses regarding the participation process (Q8). As for the ePRO system help desk (Q9), approximately 60% of responses were favorable, indicating that the issue was resolved within the expected timeframe, whereas approximately 30% were unfavorable.

**Table 5:** Summary of ePRO usability assessment by investigators (including healthcare professionals)

Question*		n/N (%)
Q3. Was the training material for ePRO system (myMedidata Patient Cloud Registration) easy to understand?		
	Favorable (very much or somewhat)**	8/24 (33.3)
Q4. Was the training for ePRO system (myMedidata Patient Cloud Registration) useful?		
	Favorable (very much or somewhat)	14/24 (58.3)
Q5. Was the material provided for explanation regarding ePRO use (informed consent document) easy to understand?		
	Favorable (very much or somewhat)	8/23 (34.8)
Q6. Was the material provided for explanation regarding ePRO use (informed consent document) useful?		
	Favorable (very much or somewhat)	14/24 (58.3)
Q7. Was the explanation regarding ePRO use smoothly done?		
	Favorable (very much or somewhat)	11/23 (47.8)
Q8. Was the participation process for ePRO use (from participant registration using EDC to e-mail delivery for ePRO account activation) done smoothly?		
	Favorable (very much or somewhat)	7/22 (31.8)
Q9. Was the issue of ePRO system (myMedidata Patient Cloud Registration) resolved within the expected timeframe at the helpdesk?		
	Favorable (very much or somewhat)	11/19 (57.9)

*Q1 and Q2 (information on sites and respondents) were outside the scope of the analysis

**Assessed in 5 grades: Very much / Somewhat / Neutral / Not so much / Not at all

## Discussion

ePRO offers advantages in reducing burden, boosting compliance, and improving the quality of captured data compared to paper PRO. Additionally, reminder notifications could help prevent participants from missing information entry in the study. The use of ePRO may lead to more consistent collection, management, and evaluation of data in accordance with the ALCOA principles (*Attributable*, *Legible*, *Contemporaneous*, *Original*, *Accurate*), which are fundamental elements of data integrity as referenced by regulatory authorities such as the Food and Drug Administration (FDA) and European Medicines Agency (EMA) in the guidance documents [8, 9]. PRO provides valuable insights into not only clinical trials but also PMS, specifically DUS. While provisioned devices are common in many clinical trials, the BYOD approach is more practical in DUS that often require more investigational sites and participants.

Several studies have explored the compatibility of BYOD and other PRO formats. Byrom et al. [10] examined the measurement comparability of three PRO formats (paper, provisioned device, and BYOD) in 155 patients with chronic pain, focusing on a single PRO completion at a study visit. Another observational cross-over study involving 64 participants with chronic obstructive pulmonary disease (COPD) quantitatively evaluated the comparability of PRO measurements between the provisioned device and BYOD used daily for 15 days at home. High measure completion rates were observed for both device types, with equivalent scores between the two formats, supporting the use of BYOD in collecting PRO data across demographically diverse patient populations [11]. Qualitative evaluations were also conducted covering 64 participants with COPD to reveal minimal differences in participants’ experiences when completing PRO measures on provisioned devices versus BYOD [12]. The ISPOR’s (also known as *The Professional Society for Health Economics and Outcomes Research*) updated task force report provides best practice recommendations based on the current body of evidence and technological advancements related to measurement comparability between PRO data collection modes [3]. The report concludes that further testing of measurement comparability between the data collection modes is unnecessary where sufficient evidence of measurement comparability and best practices for faithful migration exist, including scenarios such as “mixing modes” within clinical trials, such as BYOD designs.

In Japan, Naiki et al. [13] conducted a single-center prospective observational study involving 40 participants with advanced cancer undergoing chemotherapy at an outpatient clinic to assess the feasibility of using ePRO with BYOD for detecting adverse events. Their findings suggest that integrating ePRO into outpatient care could help clinicians in promptly identifying adverse events at earlier stages. In a multicenter exploratory study aimed at assessing the feasibility of a communication tool using social networking service (SNS) for collecting PRO data from 73 patients with breast cancer [14], patients’ acceptance of the system was positive, with a satisfactory response rate. Moreover, the number of PROs obtained from older patients was comparable to or even higher than that of younger patients, indicating that the system was effectively designed based on daily-use applications.

The analysis results of this study indicate that collecting information with ePRO is comparable to using paper PRO. There was no major difference in data entry status between ePRO and paper PRO. Although the results of subgroup analyses should be interpreted with caution because the subgroup analyses were exploratory in nature, no trend toward a higher proportion of missing data with ePRO regardless of category, such as sex or age (non-older adults and older adults).

The high proportion of missing data on Day 1 with ePRO was due to a delay in starting ePRO data entry attributable to the time taken for account activation. Similarly, there was a possibility of incorrect or missing entry of the observation start date, contributing to the high proportion of missing data on Day 1 and Day 7 with paper PRO. The higher proportion of missing data with ePRO in Group 1 than in Group 2 found in Periods 1 and 2 could be due to the time taken to activate the ePRO account. Sensitivity analyses suggested that the increase in missing data could be due to the failure of the alert e-mail delivery. Although very little data were deemed unevaluable, it would be required to adopt certain remedial measures at DUS in the future, such as automatically supplementing information on the ePRO system, simplifying and clarifying data entry processes, implementing selection options, and enhancing guidance on data entry.

The ePRO usability assessment results in this study do not necessarily indicate that participants facing similar situations in the future will face no difficulty in ePRO data entry as in this study. Therefore, it would be necessary to take measures to maintain participants’ motivation to enter their information. Less than half of the participants responded that e-mail alerts helped in preventing missing data entry, indicating that about half of the participants could continue data entry even without e-mail alerts if data entry could be initiated successfully on Day 1. While investigators found the training and informed consent documents for the ePRO system useful, there is still room for improvement in making these materials easier to understand. Simplifying the starting process for ePRO use appears necessary to facilitate smoother proceedings for investigators. As regards the ePRO help desk, although it could be improved depending on the issue, its usefulness was demonstrated by the many favorable responses as well as the issues that could be resolved to a certain degree.

This study clearly shows the benefit of collecting information with ePRO. For example, partially missing data could be prevented by using appropriate input formats, and errors or omissions of the observation start date could be avoided, unlike with paper PRO. Information could be collected in a timely and convenient manner from participants through well-considered enhancement for data entry. However, when participant enrollment and the start date of observation coincide, as in this study, the investigational sites and participants need to activate their ePRO account immediately and start data entry with ePRO on the enrollment day. This requirement underscores the importance of prompt account activation when introducing ePRO in actual DUS because the proportion of missing data on Day 1 with ePRO in this study was high. The burden of the account activation process in investigational sites should be reduced as much as possible for ePRO using BYOD in the future. Additionally, there is a need for reasonable data entry windows depending on the investigation items and objectives as well as operational measures to further improve the training material and support system through help desks. Collecting information from participants with ePRO will be meaningful in DUS if these measures are appropriately implemented.

### Limitations

This study had some limitations. First, only sites (mostly clinics) and investigators who thoroughly understood the objectives and demonstrated cooperative intentions were approached and selected, and only cooperative participants were enrolled. The increase in missing data could be attributed to the failure of alert e-mail delivery, as identified in the sensitivity analysis. Additionally, the collected items were not related to specific diseases or medical products, and the number of items was limited. Moreover, the observation period requiring participants’ continuous data entry was relatively short. Therefore, we need to note, considering generalization, that the environment or situations in this study may not necessarily reflect those in actual DUS, which typically entails the recording of various symptoms and assessment results from the start of medical product administration.

## Conclusion

Our two-week pilot indicates that ePRO can achieve data completeness comparable to paper in motivated clinics. This preliminary study could identify the key considerations for implementing ePRO in actual PMS, specifically DUS, thus achieving the objective of the study. Future studies need to consider the assessment of actual DUS in compliance with GPSP to address matters not considered in this preliminary study.

## Supplementary Information

Below is the link to the electronic supplementary material.

Supplementary Material 1

Supplementary Material 2

Supplementary Material 3

## Data Availability

The data that support the findings of this study are not openly available due to reasons of sensitivity.
