# Supplementary material for: A Preliminary Study Introducing Electronic Patient-Reported Outcome (ePRO) Using Bring Your Own Device (BYOD) in Post-marketing Surveillance in Japan
Source: Ther Innov Regul Sci. 2025 Sep 24;60(1):199–209. doi: 10.1007/s43441-025-00873-0 (PMC12753534; doi:10.1007/s43441-025-00873-0)
Supplement: Supplementary file 1 — Supplementary Material 1 [file 43441_2025_873_MOESM1_ESM.pdf]

Supplementary Material 1
ePRO screenshot

A Preliminary Study Introducing Electronic Patient-Reported Outcome (ePRO) Using Bring Your Own Device (BYOD) in Post-marketing Surveillance in Japan

Therapeutic Innovation & Regulatory Science

Naomi Sugimoto[1], Mika Morimasa[1], Hidetoshi Misawa[2], Nobushige Matsuoka[2], Yurami Sato[3], Hiromi Yamaguchi[1], Tetsuya Hiraiwa[2], Natsuno Yamashita[2], Akira Hoshino[2], Masanori Kawai[1]
1: PMS Affairs, Pfizer R&D Japan
2: Biometrics & Data Management, Pfizer R&D Japan
3: Pfizer Digital, Pfizer Japan Inc.
Corresponding author: Naomi Sugimoto (naomi.sugimoto@pfizer.com)

Original (English translation follows)

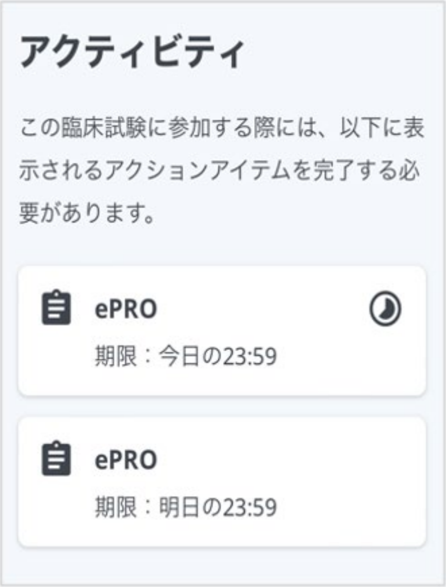

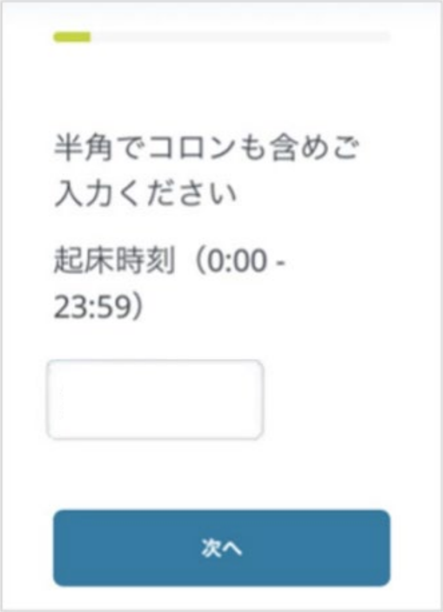

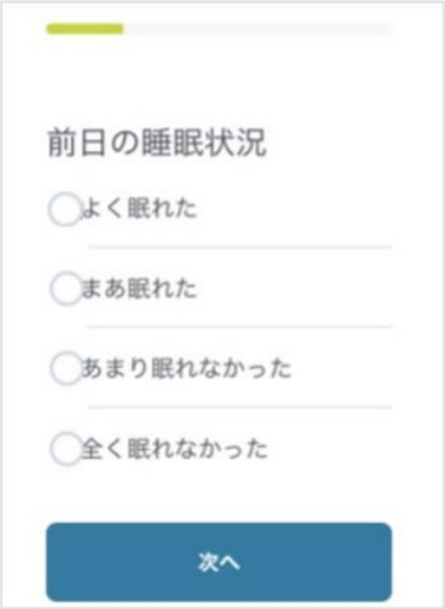
前日の睡眠状況
よく眠れた
まあ眠れた
あまり眠れなかった
全く眠れなかった
次へ

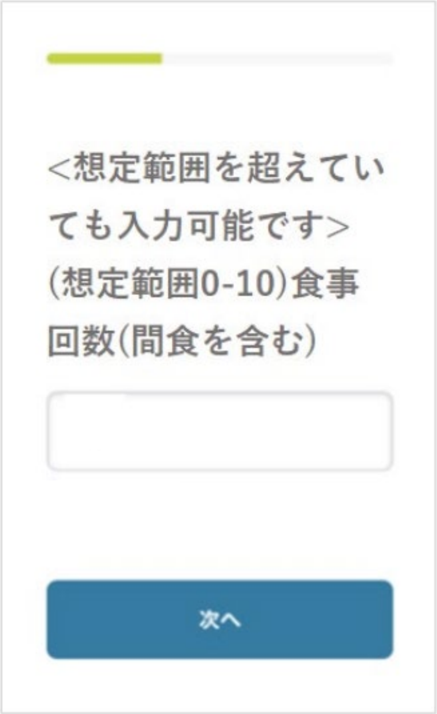
<想定範囲を超えていても入力可能です>(想定範囲0-10)食事回数(間食を含む)
次へ

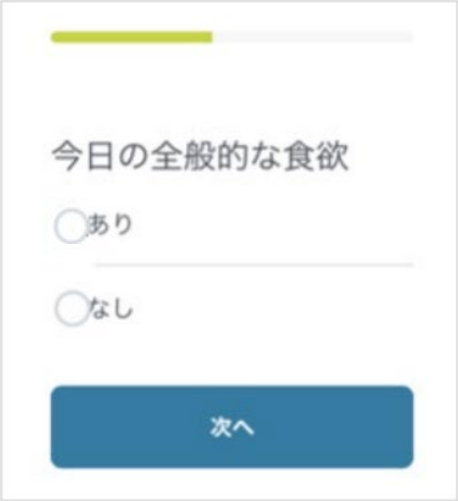
今日の全般的な食欲
あり
なし
次へ

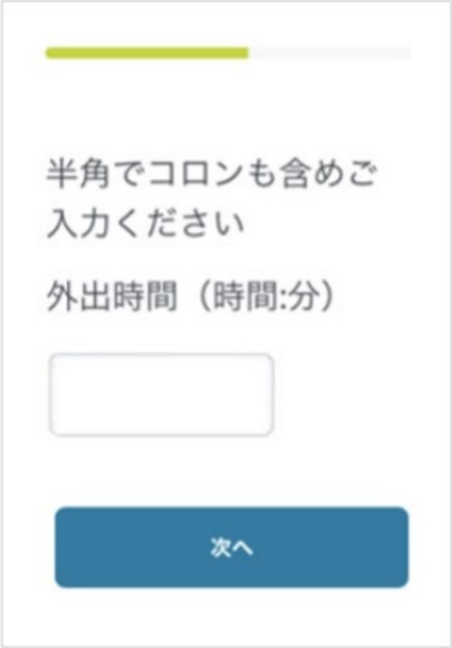
半角でコロンも含めご入力ください
外出時間（時間:分）
次へ

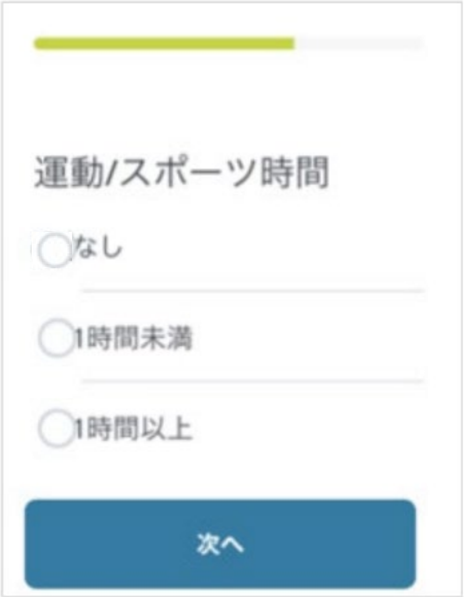

運動/スポーツ時間
なし
1時間未満
1時間以上
次へ

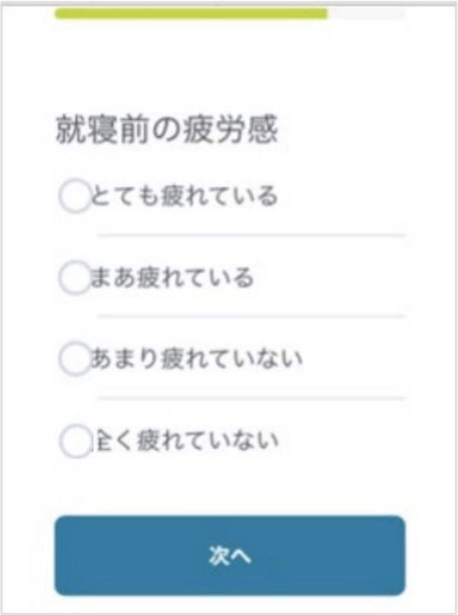

就寝前の疲労感
とても疲れている
まあ疲れている
あまり疲れていない
全く疲れていない
次へ

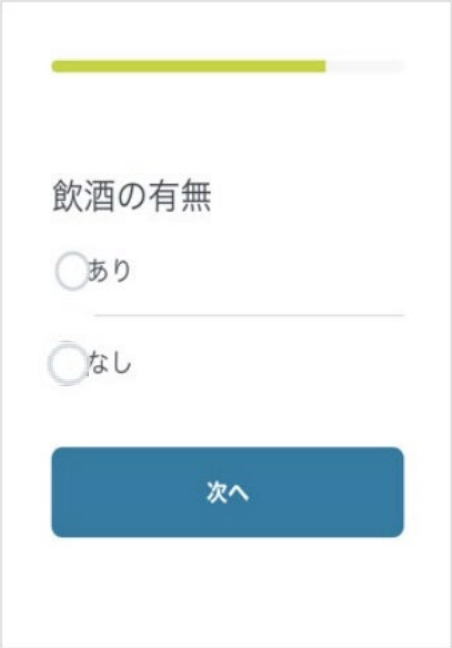
飲酒の有無
あり
なし
次へ

English translation

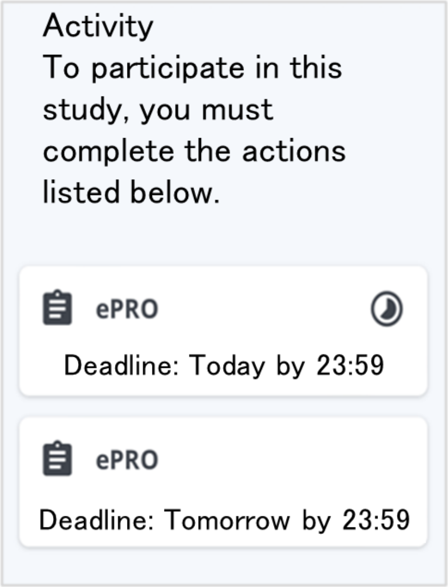

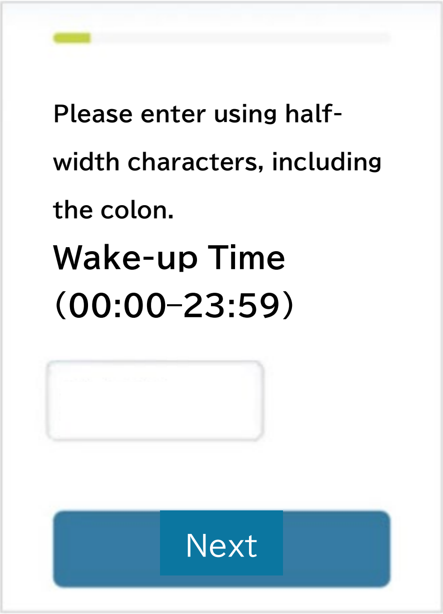

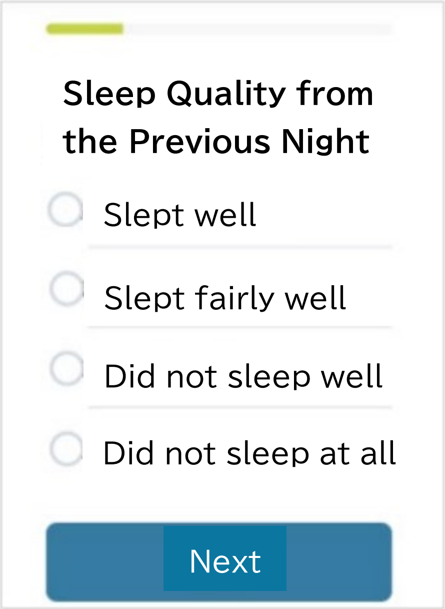
Sleep Quality from the Previous Night
Slept well
Slept fairly well
Did not sleep well
Did not sleep at all
Next

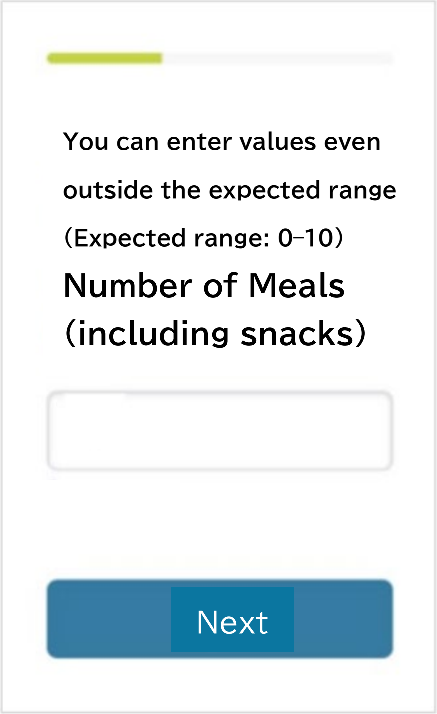
You can enter values even outside the expected range
(Expected range: 0–10)
Number of Meals (including snacks)
Next

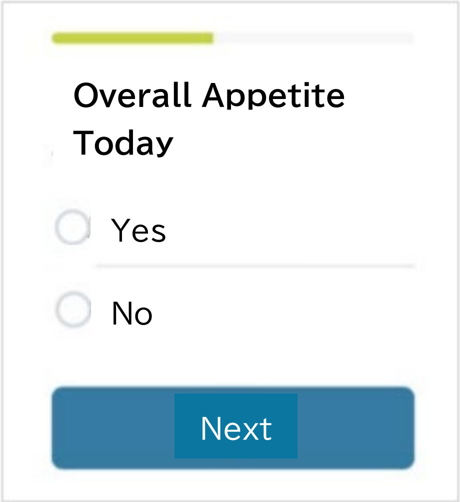
Overall Appetite Today
Yes
No
Next

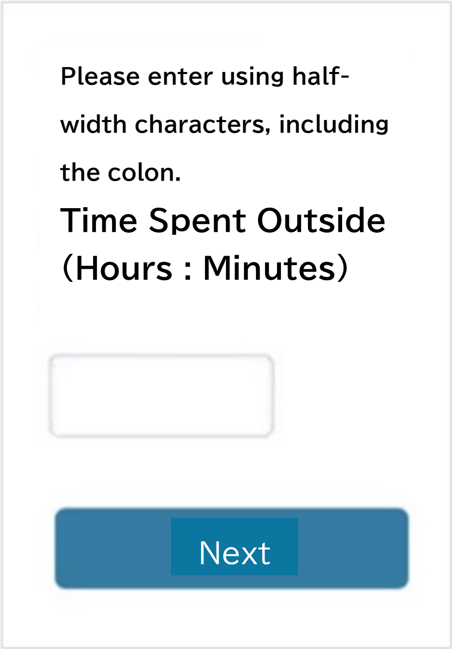
Please enter using half-width characters, including the colon.
Time Spent Outside (Hours : Minutes)
Next

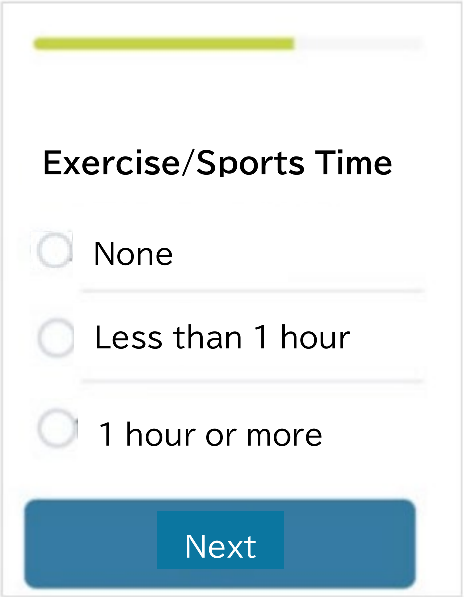
Exercise/Sports Time
None
Less than 1 hour
1 hour or more
Next

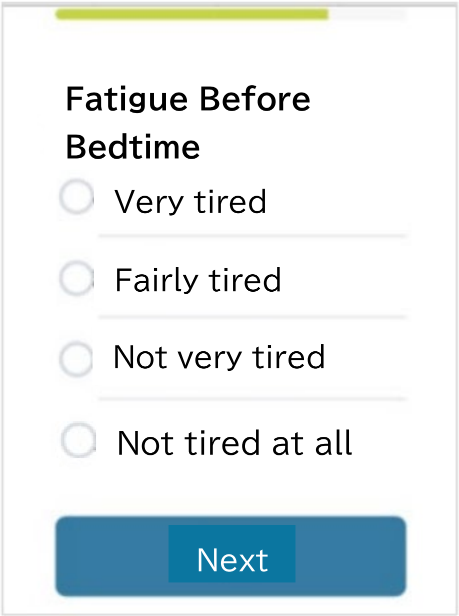
Fatigue Before Bedtime
Very tired
Fairly tired
Not very tired
Not tired at all
Next

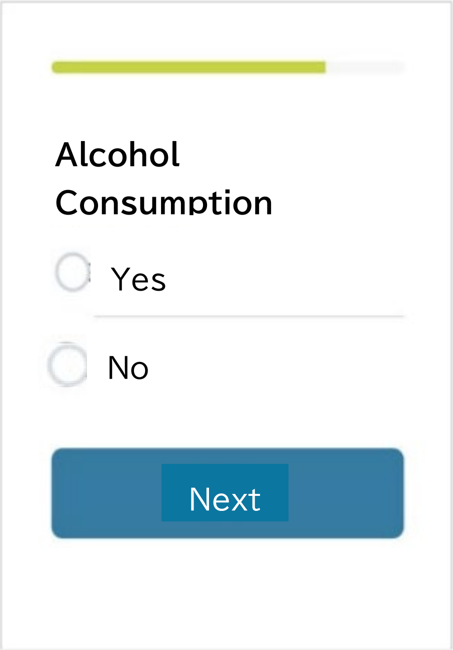
Alcohol
Consumption
Yes
No
Next
