# Supplementary material for: A Preliminary Study Introducing Electronic Patient-Reported Outcome (ePRO) Using Bring Your Own Device (BYOD) in Post-marketing Surveillance in Japan
Source: Ther Innov Regul Sci. 2025 Sep 24;60(1):199–209. doi: 10.1007/s43441-025-00873-0 (PMC12753534; doi:10.1007/s43441-025-00873-0)
Supplement: Supplementary file 2 — Supplementary Material 2 [file 43441_2025_873_MOESM2_ESM.pdf]

Supplementary Material 2

Paper Observation Diary [Sample]

A Preliminary Study Introducing Electronic Patient-Reported Outcome (ePRO) Using Bring Your Own Device (BYOD) in Post-marketing Surveillance in Japan

Therapeutic Innovation & Regulatory Science

Naomi Sugimoto[1], Mika Morimasa[1], Hidetoshi Misawa[2], Nobushige Matsuoka[2], Yurami Sato[3], Hiromi Yamaguchi[1], Tetsuya Hiraiwa[2], Natsuno Yamashita[2], Akira Hoshino[2], Masanori Kawai[1]

1: PMS Affairs, Pfizer R&D Japan

2: Biometrics & Data Management, Pfizer R&D Japan

3: Pfizer Digital, Pfizer Japan Inc.

Corresponding author: Naomi Sugimoto (naomi.sugimoto@pfizer.com)

Original (English translation follows)

紙の観察日誌【見本】

以下の点につきまして，ご協力をお願いいたします。

- ✧ 紙の観察日誌の観察開始日を１日目として、連続して７日間、毎日（遅くとも翌日までに）ご記入ください。
- ✧ 各項目について、裏面の記入例の説明に従って、回答をご記入ください。
- ✧ １枚目の医療機関保管用シート以外にご自身のお名前などの個人情報は、記入しないようご注意ください。
- ✧ ご記入いただいた情報は、この調査以外では使用しません。個人情報等のプライバシーは厳しく守られます。
- ✧ 本観察日誌の７日間の記入および PRO 使用評価票（患者用）の記入完了後、一緒にすみやかにレターパックで医療機関宛に郵送してください。

【医療機関の方へ】

- ✧ 医療機関で本観察日誌を受領後に、医療機関からファイザーにご提出いただく際には、２枚目（医療機関→ファイザー提出用シート）をミシン目に沿って切り取り、２枚目（医療機関→ファイザー提出用シート）のみをご提出ください。１枚目（医療機関保管用シート）は医療機関で必ず保管してください。

ファイザーR&D 合同会社

患者割り付け通し番号：＿＿＿＿＿＿＿＿

記入例

観察結果を記入した日付を記入ください

観察対象の日付を記入ください[医療機関から指定された本観察日誌の観察期間(7 日間の連続した日付)を記入してください]

観察日(観察対象の日付)は 1/15 であったが、当日記入を忘れ、翌日(1/16)に記入した場合の記入例です(2日以上前の観察結果は記入しないでください)

	1 日目	2 日目	3 日目	4 日目	5 日目	6 日目	7 日目
記入日	2023/ 1 /12	2023/ 1 /13	2023/ 1 / 14	2023/ 1 /16	2023/ 1 /16	2023/ 1 /17	2023/ 1 /18
観察日	2023/ 1 /12	2023/ 1 /13	2023/ 1 /14	2023/ 1 /15	2023/ 1 /16	2023/ 1 /17	2023/ 1 /18
起床時刻<br>(00:00-23:59)	6 時 30 分	6 時 30 分	6 時 30 分	7 時 00 分	6 時 50 分	7 時 15 分	7 時 00 分
前日の睡眠状況<br>(1 つ選ぶ)	☐よく眠れた<br>☑まあ眠れた<br>☐あまり眠れなかった<br>☐全く眠れなかった	☑よく眠れた<br>☐まあ眠れた<br>☐あまり眠れなかった<br>☐全く眠れなかった	☐よく眠れた<br>☑まあ眠れた<br>☐あまり眠れなかった<br>☐全く眠れなかった	☑よく眠れた<br>☐まあ眠れた<br>☐あまり眠れなかった<br>☐全く眠れなかった	☐よく眠れた<br>☑まあ眠れた<br>☐あまり眠れなかった<br>☐全く眠れなかった	☐よく眠れた<br>☐まあ眠れた<br>☑あまり眠れなかった<br>☐全く眠れなかった	☐よく眠れた<br>☐まあ眠れた<br>☑あまり眠れなかった<br>☐全く眠れなかった
食事回数<br>(間食を含む)	3 回	4 回	3 回	3 回	2 回	3 回	3 回
今日の全般的な食欲<br>(1 つ選ぶ)	☑あり<br>☐なし	☑あり<br>☐なし	☑あり<br>☐なし	☑あり<br>☐なし	☐あり<br>☑なし	☑あり<br>☐なし	☑あり<br>☐なし
外出時間	6 時間 30 分	6 時間 00 分	8 時間 00 分	2 時間 00 分	0 時間 20 分	7 時間 00 分	7 時間 00 分
運動/スポーツ時間<br>(1 つ選ぶ)	☑なし<br>☐1 時間未満<br>☐1 時間以上	☑なし<br>☐1 時間未満<br>☐1 時間以上	☑なし<br>☐1 時間未満<br>☐1 時間以上	☑なし<br>☐1 時間未満<br>☐1 時間以上	☑なし<br>☐1 時間未満<br>☐1 時間以上	☐なし<br>☑1 時間未満<br>☐1 時間以上	☐なし<br>☑1 時間未満<br>☐1 時間以上
就寝前の疲労感<br>(1 つ選ぶ)	☐とても疲れている<br>☐まあ疲れている<br>☐あまり疲れていない<br>☑全く疲れていない	☐とても疲れている<br>☐まあ疲れている<br>☐あまり疲れていない<br>☑全く疲れていない	☐とても疲れている<br>☐まあ疲れている<br>☑あまり疲れていない<br>☐全く疲れていない	☐とても疲れている<br>☐まあ疲れている<br>☐あまり疲れていない<br>☑全く疲れていない	☐とても疲れている<br>☐まあ疲れている<br>☐あまり疲れていない<br>☑全く疲れていない	☐とても疲れている<br>☐まあ疲れている<br>☐あまり疲れていない<br>☑全く疲れていない	☐とても疲れている<br>☐まあ疲れている<br>☐あまり疲れていない<br>☑全く疲れていない
飲酒の有無<br>(1 つ選ぶ)	☐あり<br>☑なし	☐あり<br>☑なし	☐あり<br>☑なし	☑あり<br>☐なし	☐あり<br>☑なし	☐あり<br>☑なし	☐あり<br>☑なし

患者割り付け通し番号：＿＿＿＿＿＿＿

以下の項目について、医療機関から指定された紙の観察日誌の観察開始期間中、毎日（遅くとも翌日までに）ご記入ください。

	1 日目	2 日目	3 日目	4 日目	5 日目	6 日目	7 日目
**記入日**	/ /	/ /	/ /	/ /	/ /	/ /	/ /
**観察日**	/ /	/ /	/ /	/ /	/ /	/ /	/ /
**起床時刻**<br>**（00:00-23:59）**	＿＿ 時 ＿＿ 分	＿＿ 時 ＿＿ 分	＿＿ 時 ＿＿ 分	＿＿ 時 ＿＿ 分	＿＿ 時 ＿＿ 分	＿＿ 時 ＿＿ 分	＿＿ 時 ＿＿ 分
**前日の睡眠状況**<br>**（1 つ選ぶ）**	□よく眠れた<br>□まあ眠れた<br>□あまり眠れなかった<br>□全く眠れなかった	□よく眠れた<br>□まあ眠れた<br>□あまり眠れなかった<br>□全く眠れなかった	□よく眠れた<br>□まあ眠れた<br>□あまり眠れなかった<br>□全く眠れなかった	□よく眠れた<br>□まあ眠れた<br>□あまり眠れなかった<br>□全く眠れなかった	□よく眠れた<br>□まあ眠れた<br>□あまり眠れなかった<br>□全く眠れなかった	□よく眠れた<br>□まあ眠れた<br>□あまり眠れなかった<br>□全く眠れなかった	□よく眠れた<br>□まあ眠れた<br>□あまり眠れなかった<br>□全く眠れなかった
**食事回数**<br>**（間食を含む）**	回	回	回	回	回	回	回
**今日の全般的な食欲**<br>**（1 つ選ぶ）**	□あり<br>□なし	□あり<br>□なし	□あり<br>□なし	□あり<br>□なし	□あり<br>□なし	□あり<br>□なし	□あり<br>□なし
**外出時間**	＿＿ 時間 ＿＿ 分	＿＿ 時間 ＿＿ 分	＿＿ 時間 ＿＿ 分	＿＿ 時間 ＿＿ 分	＿＿ 時間 ＿＿ 分	＿＿ 時間 ＿＿ 分	＿＿ 時間 ＿＿ 分
**運動/スポーツ時間**<br>**（1 つ選ぶ）**	□なし<br>□1 時間未満<br>□1 時間以上	□なし<br>□1 時間未満<br>□1 時間以上	□なし<br>□1 時間未満<br>□1 時間以上	□なし<br>□1 時間未満<br>□1 時間以上	□なし<br>□1 時間未満<br>□1 時間以上	□なし<br>□1 時間未満<br>□1 時間以上	□なし<br>□1 時間未満<br>□1 時間以上
**就寝前の疲労感**<br>**（1 つ選ぶ）**	□とても疲れている<br>□まあ疲れている<br>□あまり疲れていない<br>□全く疲れていない	□とても疲れている<br>□まあ疲れている<br>□あまり疲れていない<br>□全く疲れていない	□とても疲れている<br>□まあ疲れている<br>□あまり疲れていない<br>□全く疲れていない	□とても疲れている<br>□まあ疲れている<br>□あまり疲れていない<br>□全く疲れていない	□とても疲れている<br>□まあ疲れている<br>□あまり疲れていない<br>□全く疲れていない	□とても疲れている<br>□まあ疲れている<br>□あまり疲れていない<br>□全く疲れていない	□とても疲れている<br>□まあ疲れている<br>□あまり疲れていない<br>□全く疲れていない
**飲酒の有無**<br>**（1 つ選ぶ）**	□あり<br>□なし	□あり<br>□なし	□あり<br>□なし	□あり<br>□なし	□あり<br>□なし	□あり<br>□なし	□あり<br>□なし

医療機関名、患者登録番号（医療機関から受領した本調査の関連資材が入った封筒（表）に記載された EG から始まるあなたの登録番号）およびお名前（医療機関保管用シートのみ）をご記入ください。

医療機関名		症例登録番号	EG —
患者氏名			

（1 枚目）
医療機関保管用シート

この用紙には、個人情報や体の調子に関することは記載しないでください。

患者割り付け通し番号：＿＿＿＿＿＿＿＿

以下の項目について、医療機関から指定された紙の観察日誌の観察開始期間中、毎日（遅くとも翌日までに）ご記入ください。

	1日目	2日目	3日目	4日目	5日目	6日目	7日目
**記入日**	/ /	/ /	/ /	/ /	/ /	/ /	/ /
**観察日**	/ /	/ /	/ /	/ /	/ /	/ /	/ /
**起床時刻（00:00-23:59）**	＿＿ 時 ＿＿ 分	＿＿ 時 ＿＿ 分	＿＿ 時 ＿＿ 分	＿＿ 時 ＿＿ 分	＿＿ 時 ＿＿ 分	＿＿ 時 ＿＿ 分	＿＿ 時 ＿＿ 分
**前日の睡眠状況（1つ選ぶ）**	□よく眠れた<br>□まあ眠れた<br>□あまり眠れなかった<br>□全く眠れなかった	□よく眠れた<br>□まあ眠れた<br>□あまり眠れなかった<br>□全く眠れなかった	□よく眠れた<br>□まあ眠れた<br>□あまり眠れなかった<br>□全く眠れなかった	□よく眠れた<br>□まあ眠れた<br>□あまり眠れなかった<br>□全く眠れなかった	□よく眠れた<br>□まあ眠れた<br>□あまり眠れなかった<br>□全く眠れなかった	□よく眠れた<br>□まあ眠れた<br>□あまり眠れなかった<br>□全く眠れなかった	□よく眠れた<br>□まあ眠れた<br>□あまり眠れなかった<br>□全く眠れなかった
**食事回数（間食を含む）**	回	回	回	回	回	回	回
**今日の全般的な食欲（1つ選ぶ）**	□あり<br>□なし	□あり<br>□なし	□あり<br>□なし	□あり<br>□なし	□あり<br>□なし	□あり<br>□なし	□あり<br>□なし
**外出時間**	＿＿ 時間 ＿＿ 分	＿＿ 時間 ＿＿ 分	＿＿ 時間 ＿＿ 分	＿＿ 時間 ＿＿ 分	＿＿ 時間 ＿＿ 分	＿＿ 時間 ＿＿ 分	＿＿ 時間 ＿＿ 分
**運動/スポーツ時間（1つ選ぶ）**	□なし<br>□1時間未満<br>□1時間以上	□なし<br>□1時間未満<br>□1時間以上	□なし<br>□1時間未満<br>□1時間以上	□なし<br>□1時間未満<br>□1時間以上	□なし<br>□1時間未満<br>□1時間以上	□なし<br>□1時間未満<br>□1時間以上	□なし<br>□1時間未満<br>□1時間以上
**就寝前の疲労感（1つ選ぶ）**	□とても疲れている<br>□まあ疲れている<br>□あまり疲れていない<br>□全く疲れていない	□とても疲れている<br>□まあ疲れている<br>□あまり疲れていない<br>□全く疲れていない	□とても疲れている<br>□まあ疲れている<br>□あまり疲れていない<br>□全く疲れていない	□とても疲れている<br>□まあ疲れている<br>□あまり疲れていない<br>□全く疲れていない	□とても疲れている<br>□まあ疲れている<br>□あまり疲れていない<br>□全く疲れていない	□とても疲れている<br>□まあ疲れている<br>□あまり疲れていない<br>□全く疲れていない	□とても疲れている<br>□まあ疲れている<br>□あまり疲れていない<br>□全く疲れていない
**飲酒の有無（1つ選ぶ）**	□あり<br>□なし	□あり<br>□なし	□あり<br>□なし	□あり<br>□なし	□あり<br>□なし	□あり<br>□なし	□あり<br>□なし

医療機関名、患者登録番号（医療機関から受領した本調査の関連資材が入った封筒（表）に記載されたEGから始まるあなたの登録番号）およびお名前（医療機関保管用シートのみ）をご記入ください。

医療機関名		症例登録番号	EG —

（2枚目）
医療機関→ファイザー
提出用シート

この用紙には、個人情報や体の調子に関することは記載しないでください。

English translation

Paper Observation Diary [Sample]

Request for Your Cooperation

- ✧ Please begin recording from Day 1 of the observation period for the paper observation diary and continue for 7 consecutive days. Entries should be made daily by the following day at the latest.
- ✧ Provide response to each item according to the instructions and examples provided on the reverse side.
- ✧ Except for the first sheet (for medical institution retention), do not include any personal information such as your name.
- ✧ The information you provide will be used solely for this study. Your privacy and personal information will be strictly protected.
- ✧ After completing this paper diary for 7 days and the PRO Evaluation Form (for patients), please promptly send both documents together to the medical institution using the provided Letter Pack.

[For Medical Institutions]

- ✧ After receiving this observation diary, please detach the second sheet (for submission to Pfizer) along the perforation and submit only that sheet to Pfizer. The first sheet (for medical institution retention) must be kept at the medical institution.

Pfizer R&D Japan

Sample

Patient Assignment Serial Number：＿＿＿＿＿＿

Example Entry

Please enter the date on which the observation results were recorded.

Please enter the dates of observation (Fill in the 7 consecutive dates specified by the medical institution for this observation diary).

This is an example entry where the observation date was Jan. 15, but the entry was made the following day (Jan.16) due to a missed entry on the actual observation day. Please do not record observation results from more than two days prior.

	1 日目	2 日目	3 日目	4 日目	5 日目	6 日目	7 日目
記入日	2023/ 1 /12	2023/ 1 /13	2023/ 1 / 14	2023/ 1 /16	2023/ 1 /16	2023/ 1 /17	2023/ 1 /18
観察日	2023/ 1 /12	2023/ 1 /13	2023/ 1 /14	2023/ 1 /15	2023/ 1 /16	2023/ 1 /17	2023/ 1 /18
起床時刻 (00:00-23:59)	6 時 30 分	6 時 30 分	6 時 30 分	7 時 00 分	6 時 50 分	7 時 15 分	7 時 00 分
前日の睡眠状況 (1 つ選ぶ)	□よく眠れた<br>☑まあ眠れた<br>□あまり眠れなかった<br>□全く眠れなかった	☑よく眠れた<br>□まあ眠れた<br>□あまり眠れなかった<br>□全く眠れなかった	□よく眠れた<br>☑まあ眠れた<br>□あまり眠れなかった<br>□全く眠れなかった	☑よく眠れた<br>□まあ眠れた<br>□あまり眠れなかった<br>□全く眠れなかった	□よく眠れた<br>☑まあ眠れた<br>□あまり眠れなかった<br>□全く眠れなかった	□よく眠れた<br>□まあ眠れた<br>☑あまり眠れなかった<br>□全く眠れなかった	□よく眠れた<br>□まあ眠れた<br>☑あまり眠れなかった<br>□全く眠れなかった
食事回数 (間食を含む)	3 回	4 回	3 回	3 回	2 回	3 回	3 回
今日の全般的な食欲 (1 つ選ぶ)	☑あり<br>□なし	☑あり<br>□なし	☑あり<br>□なし	☑あり<br>□なし	□あり<br>☑なし	☑あり<br>□なし	☑あり<br>□なし
外出時間	6 時間 30 分	6 時間 00 分	8 時間 00 分	2 時間 00 分	0 時間 20 分	7 時間 00 分	7 時間 00 分
運動/スポーツ時間 (1 つ選ぶ)	☑なし<br>□1 時間未満<br>□1 時間以上	☑なし<br>□1 時間未満<br>□1 時間以上	☑なし<br>□1 時間未満<br>□1 時間以上	☑なし<br>□1 時間未満<br>□1 時間以上	☑なし<br>□1 時間未満<br>□1 時間以上	□なし<br>☑1 時間未満<br>□1 時間以上	□なし<br>☑1 時間未満<br>□1 時間以上
就寝前の疲労感 (1 つ選ぶ)	□とても疲れている<br>□まあ疲れている<br>□あまり疲れていない<br>☑全く疲れていない	□とても疲れている<br>□まあ疲れている<br>□あまり疲れていない<br>☑全く疲れていない	□とても疲れている<br>□まあ疲れている<br>☑あまり疲れていない<br>□全く疲れていない	□とても疲れている<br>□まあ疲れている<br>□あまり疲れていない<br>☑全く疲れていない	□とても疲れている<br>□まあ疲れている<br>□あまり疲れていない<br>☑全く疲れていない	□とても疲れている<br>□まあ疲れている<br>□あまり疲れていない<br>☑全く疲れていない	□とても疲れている<br>□まあ疲れている<br>□あまり疲れていない<br>☑全く疲れていない
飲酒の有無 (1 つ選ぶ)	□あり<br>☑なし	□あり<br>☑なし	□あり<br>☑なし	☑あり<br>□なし	□あり<br>☑なし	□あり<br>☑なし	□あり<br>☑なし

Patient Assignment Serial Number：______________

For the following items, please fill out the observation diary provided by the medical institution every day during the designated observation period (no later than the following day).

	Day 1	Day 2	Day 3	Day 4	Day 5	Day 6	Day 7
**Date of Entry**	/ /	/ /	/ /	/ /	/ /	/ /	/ /
Date of Observation	/ /	/ /	/ /	/ /	/ /	/ /	/ /
**Wake-up Time (00:00-23:59)**	_ _ : _ _	_ _ : _ _	_ _ : _ _	_ _ : _ _	_ _ : _ _	_ _ : _ _	_ _ : _ _
Sleep Quality from the Previous Night (Choose one)	☐Slept well<br>☐Slept fairly well<br>☐Did not sleep well<br>☐Did not sleep at all	☐Slept well<br>☐Slept fairly well<br>☐Did not sleep well<br>☐Did not sleep at all	☐Slept well<br>☐Slept fairly well<br>☐Did not sleep well<br>☐Did not sleep at all	☐Slept well<br>☐Slept fairly well<br>☐Did not sleep well<br>☐Did not sleep at all	☐Slept well<br>☐Slept fairly well<br>☐Did not sleep well<br>☐Did not sleep at all	☐Slept well<br>☐Slept fairly well<br>☐Did not sleep well<br>☐Did not sleep at all	☐Slept well<br>☐Slept fairly well<br>☐Did not sleep well<br>☐Did not sleep at all
Number of Meals (including snacks)	Times	Times	Times	Times	Times	Times	Times
Overall Appetite Today (Choose one)	☐Yes<br>☐No	☐Yes<br>☐No	☐Yes<br>☐No	☐Yes<br>☐No	☐Yes<br>☐No	☐Yes<br>☐No	☐Yes<br>☐No
Time Spent Outside	_ _Hr _ _Min	_ _Hr _ _Min	_ _Hr _ _Min	_ _Hr _ _Min	_ _Hr _ _Min	_ _Hr _ _Min	_ _Hr _ _Min
**Exercise/Sports Time (Choose one)**	☐None<br>☐Less than 1 hour<br>☐1 hour or more	☐None<br>☐Less than 1 hour<br>☐1 hour or more	☐None<br>☐Less than 1 hour<br>☐1 hour or more	☐None<br>☐Less than 1 hour<br>☐1 hour or more	☐None<br>☐Less than 1 hour<br>☐1 hour or more	☐None<br>☐Less than 1 hour<br>☐1 hour or more	☐None<br>☐Less than 1 hour<br>☐1 hour or more
**Fatigue Before Bedtime (Choose one)**	☐Very tired<br>☐Fairly tired<br>☐Not very tired<br>☐Not tired at all	☐Very tired<br>☐Fairly tired<br>☐Not very tired<br>☐Not tired at all	☐Very tired<br>☐Fairly tired<br>☐Not very tired<br>☐Not tired at all	☐Very tired<br>☐Fairly tired<br>☐Not very tired<br>☐Not tired at all	☐Very tired<br>☐Fairly tired<br>☐Not very tired<br>☐Not tired at all	☐Very tired<br>☐Fairly tired<br>☐Not very tired<br>☐Not tired at all	☐Very tired<br>☐Fairly tired<br>☐Not very tired<br>☐Not tired at all
Alcohol Consumption (Choose one)	☐Yes<br>☐No	☐Yes<br>☐No	☐Yes<br>☐No	☐Yes<br>☐No	☐Yes<br>☐No	☐Yes<br>☐No	☐Yes<br>☐No

Please fill in the name of the medical institution, your patient registration number (the number starting with 'EG' as indicated on the front of the envelope containing the study materials received from the medical institution), and your name (only on the sheet for medical institution retention).。

Medical Institution Name		Patient Registration Number	EG —
Patient Name			

(First Sheet)
Copy for Medical Institution Records

Please do not write any personal information or details about your physical condition on this form.

For the following items, please fill out the observation diary provided by the medical institution every day during the designated observation period (no later than the following day).

Sample

Patient Assignment Serial Number：＿＿＿＿＿＿＿

	Day 1	Day 2	Day 3	Day 4	Day 5	Day 6	Day 7
**Date of Entry**	/ /	/ /	/ /	/ /	/ /	/ /	/ /
**Date of Observation**	/ /	/ /	/ /	/ /	/ /	/ /	/ /
**Wake-up Time (00:00–23:59)**	_ _ : _ _	_ _ : _ _	_ _ : _ _	_ _ : _ _	_ _ : _ _	_ _ : _ _	_ _ : _ _
**Sleep Quality from the Previous Night (Choose one)**	☐Slept well<br>☐Slept fairly well<br>☐Did not sleep well<br>☐Did not sleep at all	☐Slept well<br>☐Slept fairly well<br>☐Did not sleep well<br>☐Did not sleep at all	☐Slept well<br>☐Slept fairly well<br>☐Did not sleep well<br>☐Did not sleep at all	☐Slept well<br>☐Slept fairly well<br>☐Did not sleep well<br>☐Did not sleep at all	☐Slept well<br>☐Slept fairly well<br>☐Did not sleep well<br>☐Did not sleep at all	☐Slept well<br>☐Slept fairly well<br>☐Did not sleep well<br>☐Did not sleep at all	☐Slept well<br>☐Slept fairly well<br>☐Did not sleep well<br>☐Did not sleep at all
**Number of Meals (including snacks)**	Times	Times	Times	Times	Times	Times	Times
**Overall Appetite Today (Choose one)**	☐Yes<br>☐No	☐Yes<br>☐No	☐Yes<br>☐No	☐Yes<br>☐No	☐Yes<br>☐No	☐Yes<br>☐No	☐Yes<br>☐No
**Time Spent Outside**	_ _Hr _ _Min	_ _Hr _ _Min	_ _Hr _ _Min	_ _Hr _ _Min	_ _Hr _ _Min	_ _Hr _ _Min	_ _Hr _ _Min
**Exercise/Sports Time (Choose one)**	☐None<br>☐Less than 1 hour<br>☐1 hour or more	☐None<br>☐Less than 1 hour<br>☐1 hour or more	☐None<br>☐Less than 1 hour<br>☐1 hour or more	☐None<br>☐Less than 1 hour<br>☐1 hour or more	☐None<br>☐Less than 1 hour<br>☐1 hour or more	☐None<br>☐Less than 1 hour<br>☐1 hour or more	☐None<br>☐Less than 1 hour<br>☐1 hour or more
**Fatigue Before Bedtime (Choose one)**	☐Very tired<br>☐Fairly tired<br>☐Not very tired<br>☐Not tired at all	☐Very tired<br>☐Fairly tired<br>☐Not very tired<br>☐Not tired at all	☐Very tired<br>☐Fairly tired<br>☐Not very tired<br>☐Not tired at all	☐Very tired<br>☐Fairly tired<br>☐Not very tired<br>☐Not tired at all	☐Very tired<br>☐Fairly tired<br>☐Not very tired<br>☐Not tired at all	☐Very tired<br>☐Fairly tired<br>☐Not very tired<br>☐Not tired at all	☐Very tired<br>☐Fairly tired<br>☐Not very tired<br>☐Not tired at all
**Alcohol Consumption (Choose one)**	☐Yes<br>☐No	☐Yes<br>☐No	☐Yes<br>☐No	☐Yes<br>☐No	☐Yes<br>☐No	☐Yes<br>☐No	☐Yes<br>☐No

Please fill in the name of the medical institution, your patient registration number (the number starting with 'EG' as indicated on the front of the envelope containing the study materials received from the medical institution), and your name (only on the sheet for medical institution retention).

Medical Institution Name		Patient Registration Number	EG —

(Second Sheet)
Copy for Submission to Pfizer

Please do not write any personal information or details about your physical condition on this form.

Sample
