# Supplementary material for: A Preliminary Study Introducing Electronic Patient-Reported Outcome (ePRO) Using Bring Your Own Device (BYOD) in Post-marketing Surveillance in Japan
Source: Ther Innov Regul Sci. 2025 Sep 24;60(1):199–209. doi: 10.1007/s43441-025-00873-0 (PMC12753534; doi:10.1007/s43441-025-00873-0)
Supplement: Supplementary file 3 — Supplementary Material 3 [file 43441_2025_873_MOESM3_ESM.pdf]

Supplementary Material 3

Supplementary Tables

A Preliminary Study Introducing Electronic Patient-Reported Outcome (ePRO) Using Bring Your Own Device (BYOD) in Post-marketing Surveillance in Japan

Therapeutic Innovation & Regulatory Science

Naomi Sugimoto[1], Mika Morimasa[1], Hidetoshi Misawa[2], Nobushige Matsuoka[2], Yurami Sato[3], Hiromi Yamaguchi[1], Tetsuya Hiraiwa[2], Natsuno Yamashita[2], Akira Hoshino[2], Masanori Kawai[1]

1: PMS Affairs, Pfizer R&D Japan

2: Biometrics & Data Management, Pfizer R&D Japan

3: Pfizer Digital, Pfizer Japan Inc.

Corresponding author: Naomi Sugimoto (naomi.sugimoto@pfizer.com)

Table S1 Proportion of missing data by PRO collection method: subgroup analysis

	N	ePRO n (%)	Paper PRO n (%)
Total	151	65 (43.0)	77 (51.0)
Male	59	25 (42.4)	29 (49.2)
Female	92	40 (43.5)	48 (52.2)
Age < 65	114	45 (39.5)	54 (47.4)
≥ 65	37	20 (54.1)	23 (62.2)
Age < 20	3	2 (66.7)	1 (33.3)
≥ 20, < 30	19	7 (36.8)	7 (36.8)
≥ 30, < 40	20	6 (30.0)	9 (45.0)
≥ 40, < 50	24	8 (33.3)	9 (37.5)
≥ 50, < 60	33	17 (51.5)	19 (57.6)
≥ 60	52	25 (48.1)	32 (61.5)

Table S2 Proportion of missing data by PRO collection method in each time point

	Period 1		Period 2	
Time point	ePRO in Group 1 (N=78) n (%)	Paper PRO in Group 2 (N=73) n (%)	ePRO in Group 2 (N=73) n (%)	Paper PRO in Group 1 (N=78) n (%)
Overall (Day 1 - 7)	39 (50.0)	38 (52.1)	26 (35.6)	39 (50.0)
Day 1	24 (30.8)	16 (21.9)	23 (31.5)	22 (28.2)
Day 2	17 (21.8)	10 (13.7)	18 (24.7)	16 (20.5)
Day 3	12 (15.4)	12 (16.4)	16 (21.9)	16 (20.5)
Day 4	12 (15.4)	14 (19.2)	17 (23.3)	14 (17.9)
Day 5	13 (16.7)	14 (19.2)	15 (20.5)	13 (16.7)
Day 6	14 (17.9)	14 (19.2)	16 (21.9)	15 (19.2)
Day 7	11 (14.1)	23 (31.5)	16 (21.9)	23 (29.5)

Period 1: 7 days in earlier half of the observation period, Period 2: 7 days in latter half of the observation period

Table S3 Days with missing data by PRO collection method

	ePRO (N=151)	Paper PRO (N=151)
Mean: days (SD)	1.5 (2.39)	1.5 (2.15)
Median: days (Range)	0.0 (0-7)	1.0 (0-7)
	n (%)	n (%)
0 day	86 (57.0)	74 (49.0)
1 day	25 (16.6)	32 (21.2)
2 days	9 (6.0)	15 (9.9)
3 days	4 (2.6)	6 (4.0)
4 days	3 (2.0)	4 (2.6)
5 days	4 (2.6)	6 (4.0)
6 days	3 (2.0)	2 (1.3)
7 days	17 (11.3)	12 (7.9)

Table S4 Days with missing data by PRO collection method: subgroup analysis

	N	ePRO		Paper PRO	
		Mean days (SD)	Median days (Range)	Mean days (SD)	Median days (Range)
Total	151	1.5 (2.39)	0.0 (0-7)	1.5 (2.15)	1.0 (0-7)
Male	59	1.2 (2.04)	0.0 (0-7)	1.4 (2.13)	0.0 (0-7)
Female	92	1.7 (2.58)	0.0 (0-7)	1.5 (2.17)	1.0 (0-7)
Age < 65	114	1.3 (2.19)	0.0 (0-7)	1.4 (2.13)	0.0 (0-7)
≥ 65	37	2.1 (2.84)	1.0 (0-7)	1.6 (2.23)	1.0 (0-7)
Age < 20	3	1.0 (1.00)	1.0 (0-2)	0.3 (0.58)	0.0 (0-1)
≥ 20, < 30	19	1.1 (1.72)	0.0 (0-5)	1.2 (2.12)	0.0 (0-7)
≥ 30, < 40	20	0.5 (0.89)	0.0 (0-3)	0.9 (1.33)	0.0 (0-5)
≥ 40, < 50	24	0.8 (1.62)	0.0 (0-7)	1.5 (2.43)	0.0 (0-7)
≥ 50, < 60	33	2.3 (3.02)	1.0 (0-7)	1.8 (2.31)	1.0 (0-7)
≥ 60	52	1.8 (2.67)	0.0 (0-7)	1.6 (2.23)	1.0 (0-7)

Table S5 Days with missing data by PRO collection method in each 7-day observation period

	Period 1		Period 2	
	ePRO in Group 1 (N=78)	Paper PRO in Group 2 (N=73)	ePRO in Group 2 (N=73)	Paper PRO in Group 1 (N=78)
Mean: days (SD)	1.3 (2.04)	1.4 (2.03)	1.7 (2.71)	1.5 (2.27)
Median: days (Range)	0.5 (0-7)	1.0 (0-7)	0.0 (0-7)	0.5 (0-7)
	(n, %)	(n, %)	(n, %)	(n, %)
0 day	39 (50.0)	35 (47.9)	47 (64.4)	39 (50.0)
1 day	21 (26.9)	16 (21.9)	4 (5.5)	16 (20.5)
2 days	3 (3.8)	8 (11.0)	6 (8.2)	7 (9.0)
3 days	4 (5.1)	3 (4.1)	0	3 (3.8)
4 days	2 (2.6)	3 (4.1)	1 (1.4)	1 (1.3)
5 days	2 (2.6)	3 (4.1)	2 (2.7)	3 (3.8)
6 days	3 (3.8)	0	0	2 (2.6)
7 days	4 (5.1)	5 (6.8)	13 (17.8)	7 (9.0)

Table S6 Consecutive days of evaluable data by PRO collection method

	ePRO (N=151)	Paper PRO (N=151)
Mean: days (SD)	4.5 (3.19)	4.5 (3.01)
Median: days (Range)	7.0 (0-7)	6.0 (0-7)
	n (%)	n (%)
0 day	47 (31.1)	38 (25.2)
1 day	1 (0.7)	2 (1.3)
2 days	2 (1.3)	8 (5.3)
3 days	2 (1.3)	5 (3.3)
4 days	3 (2.0)	3 (2.0)
5 days	6 (4.0)	5 (3.3)
6 days	4 (2.6)	16 (10.6)
7 days	86 (57.0)	74 (49.0)

Table S7 Consecutive days of evaluable data by PRO collection method: subgroup analysis

	N	ePRO		Paper PRO	
		Mean Days (SD)	Median Days (Range)	Mean Days (SD)	Median Days (Range)
Total	151	4.5 (3.19)	7.0 (0-7)	4.5 (3.01)	6.0 (0-7)
Male	59	4.6 (3.12)	7.0 (0-7)	4.5 (3.06)	7.0 (0-7)
Female	92	4.4 (3.26)	7.0 (0-7)	4.5 (2.99)	6.0 (0-7)
Age < 65	114	4.7 (3.13)	7.0 (0-7)	4.6 (3.01)	7.0 (0-7)
≥ 65	37	3.8 (3.34)	5.0 (0-7)	4.3 (3.02)	6.0 (0-7)
Age < 20	3	5.3 (2.08)	6.0 (3-7)	4.7 (4.04)	7.0 (0-7)
≥ 20, < 30	19	4.4 (3.47)	7.0 (0-7)	5.3 (2.75)	7.0 (0-7)
≥ 30, < 40	20	5.4 (2.80)	7.0 (0-7)	5.4 (2.44)	7.0 (0-7)
≥ 40, < 50	24	5.5 (2.62)	7.0 (0-7)	5.2 (2.75)	7.0 (0-7)
≥ 50, < 60	33	3.6 (3.46)	4.0 (0-7)	3.4 (3.35)	2.0 (0-7)
≥ 60	52	4.3 (3.26)	7.0 (0-7)	4.3 (3.00)	6.0 (0-7)

Table S8 Consecutive days of evaluable data by PRO collection method in each 7-day observation period

	Period 1		Period 2	
	ePRO in Group 1 (N=78)	Paper PRO in Group 2 (N=73)	ePRO in Group 2 (N=73)	Paper PRO in Group 1 (N=78)
Mean: days (SD)	4.3 (3.14)	4.6 (2.89)	4.7 (3.26)	4.4 (3.12)
Median: days (Range)	6.5 (0-7)	6.0 (0-7)	7.0 (0-7)	6.5 (0-7)
	n (%)	n (%)	n (%)	n (%)
0 day	24 (30.8)	16 (21.9)	23 (31.5)	22 (28.2)
1 day	1 (1.3)	0	0	2 (2.6)
2 days	1 (1.3)	5 (6.8)	1 (1.4)	3 (3.8)
3 days	2 (2.6)	4 (5.5)	0	1 (1.3)
4 days	3 (3.8)	2 (2.7)	0	1 (1.3)
5 days	4 (5.1)	2 (2.7)	2 (2.7)	3 (3.8)
6 days	4 (5.1)	9 (12.3)	0	7 (9.0)
7 days	39 (50.0)	35 (47.9)	47 (64.4)	39 (50.0)

Table S9 ePRO usability assessment by participants

Question	Group 1: n/N (%)	Group 2: n/N (%)	Total: n/N (%)
Q1. Was e-mail invitation for ePRO (myMedidata) received easily?			
Very much	30/78 (38.5)	27/71 (38.0)	57/149 (38.3)
Somewhat	23/78 (29.5)	18/71 (25.4)	41/149 (27.5)
Neutral	10/78 (12.8)	10/71 (14.1)	20/149 (13.4)
Not so much	8/78 (10.3)	5/71 (7.0)	13/149 (8.7)
Not at all	3/78 (3.8)	2/71 (2.8)	5/149 (3.4)
Invitation not received	4/78 (5.1)	9/71 (12.7)	13/149 (8.7)
Favorable (very much or somewhat)	53/78 (67.9)	45/71 (63.4)	98/149 (65.8)
Q2. Was account activation for ePRO (myMedidata) completed smoothly?			
Very much	22/74 (29.7)	24/61 (39.3)	46/135 (34.1)
Somewhat	27/74 (36.5)	22/61 (36.1)	49/135 (36.3)
Neutral	11/74 (14.9)	9/61 (14.8)	20/135 (14.8)
Not so much	13/74 (17.6)	3/61 (4.9)	16/135 (11.9)
Not at all	1/74 (1.4)	2/61 (3.3)	3/135 (2.2)
Account activation not completed	0	1/61 (1.6)	1/135 (0.7)
Favorable (very much or somewhat)	49/74 (66.2)	46/61 (75.4)	95/135 (70.4)
Q3. Was the material “myMedidata patient registration workflow (for participants)” easy to understand?			
Very much	17/74 (23.0)	18/60 (30.0)	35/134 (26.1)
Somewhat	30/74 (40.5)	24/60 (40.0)	54/134 (40.3)
Neutral	17/74 (23.0)	9/60 (15.0)	26/134 (19.4)
Not so much	9/74 (12.2)	6/60 (10.0)	15/134 (11.2)
Not at all	1/74 (1.4)	3/60 (5.0)	4/134 (3.0)
Favorable (very much or somewhat)	47/74 (63.5)	42/60 (70.0)	89/134 (66.4)
Q4. Was the material “Data entry manual for ePRO (myMedidata)” easy to understand?			
Very much	22/74 (29.7)	17/60 (28.3)	39/134 (29.1)
Somewhat	24/74 (32.4)	32/60 (53.3)	56/134 (41.8)
Neutral	18/74 (24.3)	5/60 (8.3)	23/134 (17.2)
Not so much	8/74 (0.8)	4/60 (6.7)	12/134 (9.0)
Not at all	2/74 (2.7)	2/60 (3.3)	4/134 (3.0)
Favorable (very much or somewhat)	46/74 (62.2)	49/60 (81.7)	95/134 (70.9)
Q5. Was data entry in ePRO (myMedidata) easy?			
Very much	27/73 (37.0)	27/59 (45.8)	54/132 (40.9)
Somewhat	25/73 (34.2)	17/59 (28.8)	42/132 (31.8)
Neutral	7/73 (9.6)	4/59 (6.8)	11/132 (8.3)
Not so much	9/73 (12.3)	6/59 (10.2)	15/132 (11.4)
Not at all	3/73 (4.1)	2/59 (3.4)	5/132 (3.8)
No data was entered.	2/73 (2.7)	3/59 (5.1)	5/132 (3.8)
Favorable (very much or somewhat)	52/73 (71.2)	44/59 (74.6)	96/132 (72.7)
Q6. Was e-mail alert for data entry in ePRO (myMedidata) helpful in avoiding missing entry? (Only participants not affected by delivery failures of alert e-mail were involved in analysis set.)			
Very much	11/47 (23.4)	8/41 (19.5)	19/88 (21.6)
Somewhat	7/47 (14.9)	16/41 (39.0)	23/88 (26.1)
Neutral	13/47 (27.7)	2/41 (4.9)	15/88 (17.0)
Not so much	9/47 (19.1)	7/41 (17.1)	16/88 (18.2)
Not at all	2/47 (4.3)	4/41 (9.8)	6/88(6.8)
No data was entered.	0	1/41 (2.4)	1/88 (1.1)
Alert e-mail was not received.	5/47 (10.6)	3/41 (7.3)	8/88 (9.1)
Favorable (very much or somewhat)	18/47 (38.3)	24/41 (58.5)	42/88 (47.7)
Q7. Was the issue resolved within the expected timeframe at “Medidata helpdesk (Patient Cloud Device Support)”?			
Very much	2/73 (2.7)	5/57 (8.8)	7/130 (5.4)
Somewhat	3/73 (4.1)	4/57 (7.0)	7/130 (5.4)
Neutral	10/73 (13.7)	5/57 (8.8)	15/130 (11.5)
Not so much	2/73 (2.7)	1/57 (1.8)	3/130 (2.3)
Not at all	3/73 (4.1)	3/57 (5.3)	6/130 (4.6)

Question		Group 1: n/N (%)	Group 2: n/N (%)	Total: n/N (%)
	Inquiry was not made.	53/73 (72.6)	39/57 (68.4)	92/130 (70.8)
	Favorable (very much or somewhat)	5/73 (6.8)	9/57 (15.8)	14/130 (10.8)
Q8. Based on experience in this study, will electronic data entry by devices such as smartphones, PCs, etc. be able to be done without issue, if you have another opportunity to record your health information in the future?				
	Very much	18/70 (25.7)	18/59 (30.5)	36/129 (27.9)
	Somewhat	27/70 (38.6)	16/59 (27.1)	43/129 (33.3)
	Neutral	12/70 (17.1)	11/59 (18.6)	23/129 (17.8)
	Not so much	7/70 (10.0)	7/59 (11.9)	14/129 (10.9)
	Not at all	6/70 (8.6)	7/59 (11.9)	13/129 (10.1)
	Favorable (very much or somewhat)	45/70 (64.3)	34/59 (57.6)	79/129 (61.2)

Number of participants: Group 1; 78, Group 2; 73, Total; 151.

Table S10 ePRO usability assessment by investigators (including healthcare professionals)

Question*	n/N (%)
Q3. Was the training material for ePRO system (myMedidata Patient Cloud Registration) easy to understand?	
Very much	5/24 (20.8)
Somewhat	3/24 (12.5)
Neutral	8/24 (33.3)
Not so much	5/24 (20.8)
Not at all	3/24 (12.5)
Favorable (very much or somewhat)	8/24 (33.3)
Q4. Was the training for ePRO system (myMedidata Patient Cloud Registration) useful?	
Very much	5/24 (20.8)
Somewhat	9/24 (37.5)
Neutral	5/24 (20.8)
Not so much	4/24 (16.7)
Not at all	1/24 (4.2)
Favorable (very much or somewhat)	14/24 (58.3)
Q5. Was the material provided for explanation regarding ePRO use (informed consent document) easy to understand?	
Very much	5/23 (21.7)
Somewhat	3/23 (13.0)
Neutral	5/23 (21.7)
Not so much	8/23 (34.8)
Not at all	2/23 (8.7)
Favorable (very much or somewhat)	8/23 (34.8)
Q6. Was the material provided for explanation regarding ePRO use (informed consent document) useful?	
Very much	7/24 (29.2)
Somewhat	7/24 (29.2)
Neutral	5/24 (20.8)
Not so much	3/24 (12.5)
Not at all	2/24 (8.3)
Favorable (very much or somewhat)	14/24 (58.3)
Q7. Was the explanation regarding ePRO use smoothly done?	
Very much	5/23 (21.7)
Somewhat	6/23 (26.1)
Neutral	2/23 (8.7)
Not so much	8/23 (34.8)
Not at all	2/23 (8.7)
Favorable (very much or somewhat)	11/23 (47.8)
Q8. Was the participation process for ePRO use (from participant registration using EDC to e-mail delivery for ePRO account activation) done smoothly?	
Very much	3/22 (13.6)
Somewhat	4/22 (18.2)
Neutral	5/22 (22.7)
Not so much	6/22 (27.3)
Not at all	4/22 (18.2)
Favorable (very much or somewhat)	7/22 (31.8)
Q9. Was the issue of ePRO system (myMedidata Patient Cloud Registration) resolved within the expected timeframe at the helpdesk?	
Very much	5/19 (26.3)
Somewhat	6/19 (31.6)
Neutral	2/19 (10.5)
Not so much	4/19 (21.1)
Not at all	2/19 (10.5)
Favorable (very much or somewhat)	11/19 (57.9)

* Q1 and Q2 (information on sites and respondents) were outside the scope of the analysis.
